# Spatio-Temporal Variation in Effects of Upwelling on the Fatty Acid Composition of Benthic Filter Feeders in the Southern Benguela Ecosystem: Not All Upwelling Is Equal

**DOI:** 10.1371/journal.pone.0161919

**Published:** 2016-08-29

**Authors:** Eleonora Puccinelli, Christopher David McQuaid, Margaux Noyon

**Affiliations:** 1 Department of Zoology and Entomology, Rhodes University, Grahamstown, South Africa; 2 Department of Oceanography, Marine Research Institute, University of Cape Town, Rondebosch 7701, Cape Town, South Africa; 3 Marine Research Institute, Department of Biological Sciences, University of Cape Town, Rondebosch 7701, Cape Town, South Africa; Helmholtz-Zentrum fur Ozeanforschung Kiel, GERMANY

## Abstract

Variability in mesoscale nearshore oceanographic conditions plays an important role in the distribution of primary production and food availability for intertidal consumers. Advection of nutrient rich waters by upwelling usually allows the proliferation of diatoms, later replaced by dinoflagellates. We examined upwelling effects on the fatty acid (FA) signature of a benthic intertidal filter feeder to identify its response to pulsed variability in food availability. The study took place in two contrasting seasons and at two upwelling and two non-upwelling sites interspersed within the southern Benguela upwelling system of South Africa. We investigated the FA composition of the adductor muscles and gonads of the mussel *Mytilus galloprovincialis* to assess how FA are apportioned to the different tissues and whether this changes between upwelling and non-upwelling conditions. *In situ* temperature loggers used to identify upwelling conditions at the four sites indicated that such events occurred only at the upwelling centres and only in summer. Tissues differed strongly, with gonads presenting a higher proportion of essential FAs. This could reflect the faster turnover rate of gonad tissue or preferential retention of specific FA for reproductive purposes. FA composition did not vary as a direct function of upwelling, but there were strong dissimilarities among sites. Upwelling influenced mussel diets at one upwelling site while at the other, the expected signature of upwelling was displaced downstream of the core of upwelling. Condition Index (CI) and Gonad Index (GI) differed among sites and were not influenced by upwelling, with GI being comparable among sites. In addition, FA proportions were consistent among sites, indicating similar food quality and quantity over time and under upwelling and non-upwelling conditions. This suggests that the influence of upwelling on the west coast of South Africa is pervasive and diffuse, rather than discrete; while nearshore retention or advection of upwelled water is critical and site-specific so that the effects of upwelling differ even among sites categorised as upwelling centres.

## Introduction

Temporal and spatial variation in mesoscale nearshore oceanographic conditions plays an important role in the distribution of primary production [1,2], resulting in differences in the availability of resources for intertidal consumers [3,4]. These differences in food availability can strongly influence the distribution and metabolism of these organisms [5–7]. One feature that can influence food availability is represented by upwelling events, which bring deep, nutrient-rich waters into coastal ecosystems, promoting nearshore phytoplankton production [8–10]. Coastal upwelling supports many of the world’s most important pelagic fisheries [11] and previous studies have shown the importance of upwelling to benthic communities through the enhancement of primary production and food availability for primary consumers [12–14]. During periods of active upwelling, primary production usually exceeds 1 g carbon m^-2^ day^-2^ and can reach up to 10 g carbon m^-2^ day^-2^ [12]. The biological consequences associated with these events are critically important for consumers in costal environments. For instance, the reproductive peak of some invertebrates has been shown to coincide with the upwelling season [15,16], enabling planktotrophic larvae to benefit from the phytoplankton rich water.

Upwelling events are highly variable in duration, intensity and frequency [17,18]. They are usually stronger and sometimes only occur during the spring and summer months [19–21], leading to marked seasonal shifts in primary production [22]. This variability has important biological consequences for the timing of energy input into the coastal system, with implications that propagate up the food chain, affecting trophic dynamics and thus ecosystem functioning [23,24]. Strong upwelling can reduce recruitment of benthic organisms due to the offshore export of larvae away from favourable settling grounds [25]. Likewise, coastal phytoplankton composition fluctuates seasonally in response to the nutrient input associated with the seasonality of upwelling [26], usually going through a succession of communities dominated first by diatoms and then dinoflagellates [27–29]. These changes at the base of the food web may cause structural changes at different scales of observation, from individuals to the whole food web, by influencing the richness and abundance of intermediate and higher trophic levels [30].

The eastern boundary current system off the west coast of Southern Africa is recognized as one of the world’s largest coastal upwelling systems [31]. It is characterized by the north flowing Benguela Current, of Antarctic origin [32,33], which is associated with strong, wind-driven upwelling events, that occur predominantly in the austral summer [34,19]. The natural seasonality of upwelling events on the west coast of South Africa provides the opportunity to investigate the relationship between temporal variability in nearshore oceanographic conditions and the diets of benthic populations. We used fatty acid (FA) analysis to examine the spatial-temporal scales at which upwelling effects are manifest in the diet of benthic filter feeders, comparing recognised upwelling centres with downstream sites, over time. The FA technique is recognized as a useful tool for understanding the trophic relationship between food source and consumer, providing information on ecosystem dynamics and functioning over a relatively short period of time [35], making it an appropriate tool for this type of study. Our main hypothesis was that filter feeder diets change directly in response to upwelling. Within this context, we investigated several aspects of these dynamics. First, we hypothesised that the FA composition of filter feeders and suspended organic matter (SPM) would be characterized by diatom FA trophic markers (FATM) at upwelling centres during, or shortly after upwelling events, while this signature should decrease downstream of these centres. Similarly, we expected the proportion of dinoflagellate biomarkers to be stronger in specimens and SPM downstream of upwelling sites, as observed by Allan et al. [36], or to increase in the period following an upwelling event, once the nutrient concentration in the water is reduced. Secondly, we expected specimens from upwelling centres to exhibit better body condition than those from non-upwelling areas as overall they are expected to encounter higher food concentrations and potentially accumulate lipids, as energy storage, for food-shortage periods. Lastly, we wanted to identify how FA composition differs between the various tissues of filter feeders in different months and in relation to upwelling.

## Materials and Methods

This study was carried out in accordance with the requirements in the ‘‘permit for the purposes of a scientific investigation or practical experiment in term of section 83 of the Marine Living Resources Act, 1988 (Act no 18 of 1998)”. This permit was approved by the Chief Director of Fisheries Research and Development; Department of Agriculture, Forestry and Fishery, Republic of South Africa (Permit ref. no: RES2012/05 and RES2013/09).

### Study area

**Fig 1:**
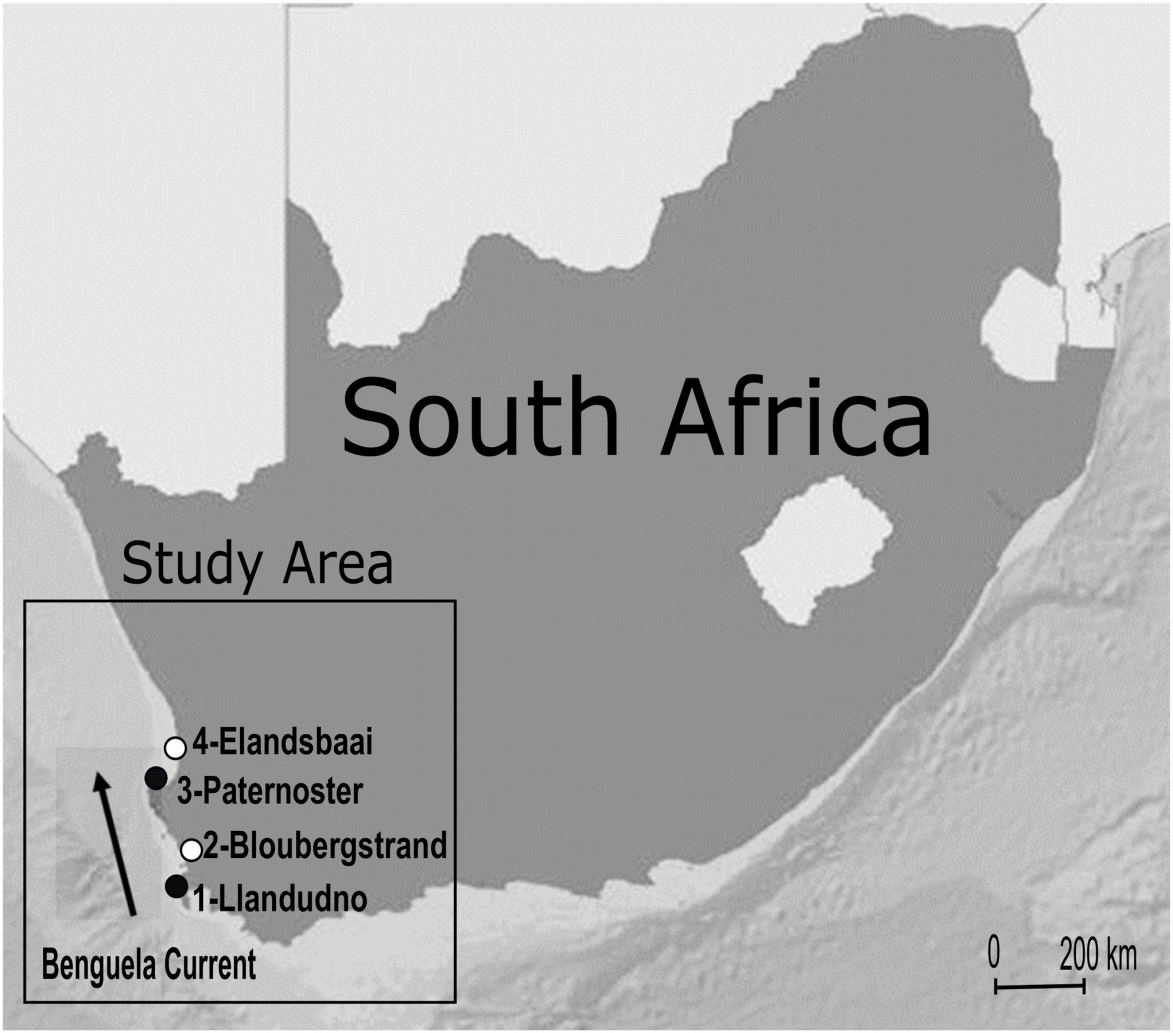
Map of the study area on the west coast of South Africa showing the sampling sites in upwelling (black) and non-upwelling (white) areas.

The study was conducted along the South African west coast (Fig 1, 34.4°–32.19°S°, 17.52°–18.27°E). The Benguela Current flows south-north along this coast and samples were collected at four sites identified by Xavier et al. as upwelling or non-upwelling centres [37]: sites 1 (Llandudno) and 3 (Paternoster) were categorised *a priori* as upwelling sites, and sites 2 (Bloubergstrand) and 4 (Elandsbaai) as non-upwelling or downstream sites (Fig 1). Sampling was carried out on four occasions: twice in austral summer (10–11^th^ December 2012 and 8–9^th^ February 2013), during the upwelling season, and twice in winter (12–13^th^ June and 8–9^th^ July 2013), during the non-upwelling season.

### Upwelling characterization

To characterize upwelling frequency and intensity, sea temperature was recorded *in situ* at each site from December 2012 to July 2013, using four temperature loggers at each site deployed intertidally during low tide. The loggers were composed of an iButton (model DS 1922L Dallas Maxim, CA, USA, Thermochron high resolution, − 40°C to + 85°C with an accuracy of ± 0.0625°C) covered with teflon and glued with two-component epoxy (Alcolin rapid-epoxy) onto perspex plates which were screwed onto the rock surface. All loggers were programmed to measure temperature every 30 min and were replaced every three months due to memory limitations. The iButtons were programmed using the software ColdChain Thermo Dynamics. Maximum tidal range on this coast is approximately two meters and the locations of loggers differed slightly among sites, but all were within the same 20 cm tidal range and deployed next to mussel beds. Hourly data of predicted tidal height at the sampling sites were provided from the website XTide: harmonic tide clock and tide predictor (http://www.flaterco.com/xtide/xtide.html), and were used to separate temperature data for periods of immersion and emersion of the loggers. Upwelling events in this region can be defined as a daily decline in mean temperature (ΔT) of ≥ 5°C [34,38], while a drop in daily mean temperature of 1–4°C was defined as a weak cooling event. The number of successive days after each cooling event in which the temperature remained constant (ΔT ≤ 5°C) from the initial drop was also recorded. The results of the temperature loggers were used to confirm the *a priori* characterization of sites as either upwelling or non-upwelling. Specifically, we used temperature data from 5 days prior a sampling event to categorize upwelling (48 measurements per day per data logger).

### Sampling

#### Fatty acids

To investigate the influence of upwelling on filter feeder diets and metabolism, we studied the adductor muscles and gonads of the mussel *Mytilus galloprovincialis*. The adductor muscle was chosen due to its low turnover rates, making it representative of a time-integrated diet [39], while the composition of the gonad provides insight into the reproductive state. On each sampling occasion and at each site, six haphazardly selected individuals were collected, dissected and processed for FA analyses. In addition, to assess if food quality differed among sites or varied among sampling occasions, three replicates of 5 L of seawater were collected from the shore to evaluate the SPM. Water samples were filtered gently (< 5 cm Hg vacuum) onto pre-combusted (450°C) GF/F filters (0.7 μm pore size and 47 mm diameter). The tissue and SPM samples were then flash frozen in liquid nitrogen and transferred to a– 80°C freezer until processing.

#### Condition and gonad indices

To understand the possible effects of upwelling on the condition of mussels, the condition index (CI) and the gonad index (GI) of *M*. *galloprovincialis* were measured from specimens at each site on each of the four sampling occasions. For the CI, 20 haphazardly selected adult mussels (4–5 cm shell length) were collected and kept frozen at– 20°C until processing. The shells and the soft body tissues of each mussel was dissected and dried at 60°C for 48 h. CI was also calculated for the samples used for the FA analyses (providing six additional individuals per site). For these samples, the soft parts were freeze dried for 24 h as drying at 60°C would degrade the FA. No differences in the CI were recorded between specimens dried with the two different methods (ANOVA, *p* > 0.05). Consequently all replicates were pooled for the CI analyses (n = 26 per site). The CI was then calculated as a percentage of the dry soft tissue weight over the dry shell weight, following Davenport and Chen [40]:

$$CI=\frac{dry\;soft\;tissue\;weight}{dry\;shell\;weight}X\;100$$

The GI was measured only for the samples used for the FA analyses (n = 6 per site). The gonads were dissected and freeze dried for 24 h. The GI was calculated as a percentage, by dividing the dry weight of the gonad by the total body dry weight following Williams and Babcock [41]:

$$GI=\frac{dry\;gonad\;weight}{dry\;total\;body\;weight}\;X\;100$$

#### Fatty acid analyses

Samples were lyophilized (VirTis BenchTop K) for 24 h, then stored at -80°C until total lipids were extracted and trans-esterified using a modified Indarti et al [42] one-step procedure within 6 months of collection. A known amount of tissue (between 25 and 50 mg of dry weight) for each sample was homogenized into a 4 mL fresh solution of a mixture of methanol, concentrated sulphuric acid and chloroform containing 0.01% of an anti-oxidant, BHT (butylated hydroxytoluene) (1.7/0.3/2.0 v/v/v), and closed under nitrogen. The extraction and transesterification reactions occurred at 100°C for 30 minutes. The FA methyl esters (FAME) formed were then stored at -80°C until Gas Chromatography (GC) analyses. FAME composition of each sample was determined by GC (Agilent Technologies 7890A) equipped with a ZB-Waxplus capillary column (ZB-Waxplus 320 column), with helium as the carrier gas at a flow rate of 1.664 ml min^−1^. The injector was at a temperature of 250°C. The flame ionization detector was set at 260°C, and the oven was initially set at 70°C. After 1 min, the oven temperature was increased by 40°C min^-1^ until 170°C and then raised to 250°C at a rate of 2.5°C min^−1^ and held for 4.5 min. Peaks were integrated using GC ChemStation software (Agilent Technologies, version B.04.02), identified by comparison with retention times of external known standards (37 component fatty acid methyl ester mix Supelco, marine PUFA no. 1 Supelco, menhaden oil PUFA no. 3, bacterial acid methylesters mix Supelco), as well as by mass spectrometry analyses (Agilent Technologies 7000 GC/MS Triple Quad; Agilent Mass Hunter (MS), version B.05.00,) using the NIST library. Each FA was measured as a proportion of the total FA (TFA) composition (% by weight of TFA) and peak areas were corrected according to the FID response to FA chain length [43]. FA are reported using a shorthand notation of A:Bw*x*, where A indicates the number of carbon atoms, B is the number of double bonds and *x* indicates the position of the first double bond relative to the terminal methyl group [44].

### Data analysis

#### Upwelling characterisation

To test the *a priori* characterization of upwelling or non-upwelling conditions at the four sites, we used temperature data from 5 days prior to each sampling event at each site. Because in the month of December we sampled on the day of deployment of the data loggers, we decided not to include these data in the analyses that tested for upwelling effects, and for that month only to evaluate differences in FA composition, CI and GI of mussels among sites (see details below). To test for differences in temperature among sites over time we ran an analysis of variance (ANOVA) with a mixed model design consisting of the factors: month (three levels, fixed and crossed with the other factors), upwelling (two levels, fixed and crossed with month) and site (two levels, random and nested in upwelling). In the event of significant results, Tukey HSD *post hoc* tests were performed. The violation of homogeneity of variances was considered to be acceptable because ANOVA is relatively robust to heterogeneous variances for large designs such as the one in this study [45]. Analyses were performed using STATISTICA v12 (StatSoft).

#### Fatty acids

To investigate the FA composition of mussels and SPM in relation to upwelling, we used the categorization of upwelling and non-upwelling conditions based on the temperature logger results, which indicated upwelling only at sites 1 and 3 and only in February. For June and July, all sites were considered as representing non-upwelling conditions (see Results and Table A in S1 File). Thus we evaluated the effect of upwelling for the month of February only, using a mixed model design consisting of the factors: upwelling (two levels, fixed and crossed with tissue), site (two levels, random and nested in upwelling), and tissue (two levels, fixed and crossed with all the other factors). To test for differences among sites in June and July, we used a similar design without the factor upwelling, as conditions at all sites were considered to be non-upwelling. For this analysis, the factors were: site (four levels, random and crossed with month and tissue), month (two levels, random and crossed with site and tissue) and tissue (two levels, fixed and crossed with site and month). With the present tools we were not able to assess whether upwelling occurred in December or at which sites, thus we decided to analyse this month separately from the others and we only tested for the factor site (four levels, random). Similarly, the experimental design to test for FA differences in SPM in February comprised the factors: upwelling (two levels, fixed) and site (two levels, random and nested in upwelling). The design for the SPM in June and July consisted of the factors: site (four levels, random and crossed with month) and month (two levels, random and crossed with site); while in December the analysis comprised only the factor site (four levels, random). A Multivariate Permutation Analysis [46] of Bray-Curtis dissimilarities was used to assess differences among factors. Each term in the analysis was tested using > 9999 permutations as the relevant permutable units [47]. We used Principal Component Analysis (PCA), of square root transformed data, to explore differences in FA signatures among specimens [48]. For clarity, in the eigenvector plot we show only FA which had a coefficient higher than 0.5 for one of the axes. The combined results of the PCA and SIMPER (similarity percentage, PRIMER) were used to identify the FA responsible for differences among groups of samples, as the number of FA used (variables) was important and sometimes difficult to interpret based only on the PCA. Only FA forming more than 1% of TFA were included in the analyses (*i*.*e*. 31 FA used). The analyses were conducted using the PRIMER v6 and PERMANOVA+ [48,49].

#### Condition Index and Gonad Index

Two-way ANOVA was used to test for an effect of upwelling on the CI of *M*. *galloprovincialis* in February. This included the factors: upwelling (two levels, fixed) and site (two levels, random and nested in upwelling). A second design was used to evaluate significant differences in CI of specimens among sites for June and July consisting of: month (two levels, random) and site (four levels, random); while a third design was used for December which comprised the factor site only (four levels, random). The same designs were used to test for significant effects on the GI of specimens used for the FA analyses. The violation of homogeneity of variances was considered to be acceptable because ANOVA is relatively robust to heterogeneous variances for large designs such as the one used in this study [45].

## Results

### Upwelling characterization

**Fig 2:**
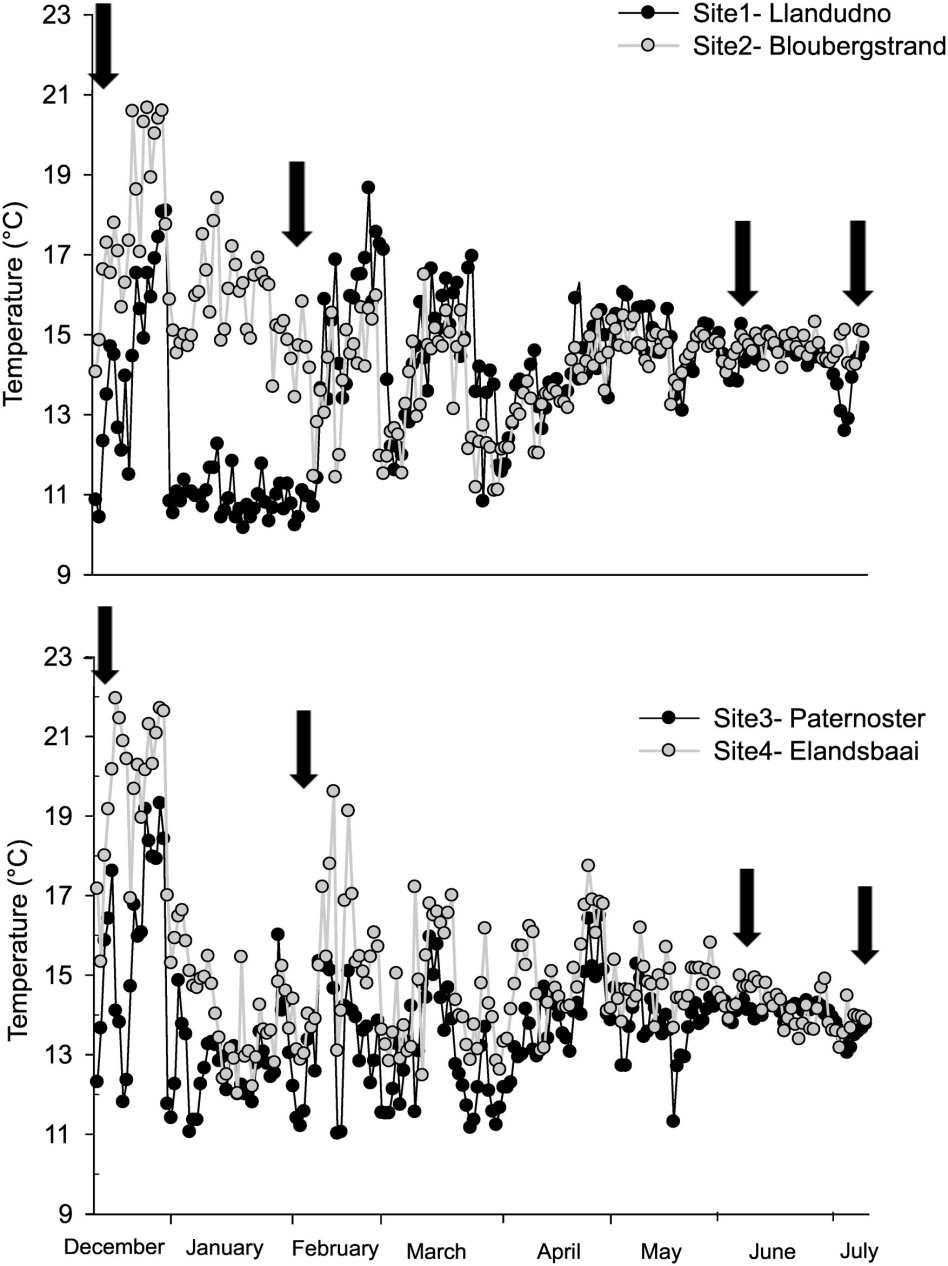
Mean daily in situ sea temperatures at four sites on the South African west coast derived from onshore loggers for the duration of the investigation. The arrows indicate the four sampling events. Upwelling events were defined by a daily decline in mean temperature (ΔT) of ≥ 5°C.

Onshore water temperature showed pronounced variability over the study period and marked differences among sites (Fig 2). From December to February conditions at the upwelling sites 1 and 3 (Llandudno and Paternoster) were colder than at their downstream sites 2 and 4 (Bloubergstrand and Elandsbaai; ANOVA, *p* < 0.01) by an average of 4.3 and 2.4°C respectively. ANOVA showed no significant differences among sites in June and July (*p* > 0.05). Periods of cooling events occurred at all sites, but with differences between the two categories of sites during the upwelling season. Sites 1 and 3 experienced three and six events of strong upwelling, respectively. One of these was protracted, running from 31^st^ Dec and involving low temperatures (10–12°C) that lasted until the 13^th^ of February (ΔT 8°C) at site 1 and until 30^th^ of January (ΔT 5°C) at site 3. The other upwelling events were shorter (6–10 days) and less intense (ΔT 5°C), occurring in February and March. At sites 2 and 4, cooling events involved a drop in temperature of about 4°C with the exception of site 4, where a decrease of 6 and then 7°C was observed on the 31^st^ December and 16^th^ February, respectively. These events were less prolonged compared to upwelling events. Based on these analyses we confirmed the occurrence of upwelling at sites 1 and 3 (identified *a priori* as upwelling sites) in February, with no upwelling during winter (June and July) at these sites or at the non-upwelling sites, 2 and 4, in any sampling month. The protracted period of low seawater temperature that was observed at site 1 in January/ February suggested that upwelling occurred throughout the period of time prior the sampling event in February. Thus, we decided to classify site 1 as an upwelling site for the February collection. Additionally, these results allowed us to treat all the sites as experiencing non-upwelling conditions during winter (see Table A in S1 File). The lack of information on water temperature prior to sampling in December, due to the deployment of the data loggers on the same day as sample collection, did not allow us to categorize sites in either upwelling or non-upwelling. Thus, the samples for December were not tested for upwelling effects.

### Fatty acid composition

#### Food source

No effect of upwelling on the SPM was found in this study, however PERMANOVA showed a significant effect of month and the interaction between month and site for June and July (both *p* < 0.01) and significant site effects in both December and February. Generally, SPM had higher proportions of saturated FA (SFA, 40–60%) than monounsaturated FA (MUFA, 20–45%) and polyunsaturated FA (PUFA, 10–25%) in all months (Table 1). The main SFA were represented by 16:0 (14–33%), followed by 18:0 (6–15%) and 14:0 (3–10%). The MUFA with the highest values were 18:1w9 (3–26%), 18:1w7 (1–11%) and 16:1w7 (12–12%), while among PUFA, only 18:2w6 and 20:3w3 counted for more than 5% of TFA at any specific site or sampling event (Table 1). Despite PERMANOVA highlighted dissimilarities, PCA and SIMPER showed no obvious pattern among sites (S1 Fig). Importantly, the differences observed in SPM did not correspond to the pattern observed in consumer tissues.

**Table 1:** Total fatty acid composition of suspended particulate matter (SPM) collected during a) December and February and b) June and July across four sites along the South African west coast. The values are percentages expressed as mean ± standard deviation (n = 3 per site).

**A**								
	**December**				**February**			
	**1**	**2**	**3**	**4**	**1**	**2**	**3**	**4**
14:0	5.28 ± 1.22	5.93 ± 0.54	3.90 ± 0.34	3.89 ± 1.04	3.06 ± 0.13	3.28 ± 1.09	3.04 ± 0.07	2.75 ± 1.00
14:1w5	0.99 ± 0.95	1.04 ± 1.12	0.29 ± 0.26	0.44 ± 0.36	0.21 ± 0.26	0.74 ± 0.82	1.53 ± 0.27	0.61 ± 0.23
16:0	20.82 ± 5.28	26.49 ± 2.33	33.01 ± 1.32	33.09 ± 0.86	20.01 ± 7.17	19.21 ± 1.41	22.41 ± 0.53	17.34 ± 5.80
16:1w7	6.36 ± 4.87	11.58 ± 1.66	2.65 ± 0.68	3.22 ± 1.68	4.01 ± 0.89	2.68 ± 0.27	6.19 ± 0.07	1.31 ± 0.53
16:1w5	3.55 ± 2.61	0.33 ± 0.21	1.84 ± 1.02	2.91 ± 0.83	0.76 ± 0.61	3.04 ± 2.07	1.96 ± 0.42	6.19 ± 1.81
17:1w7	0.77 ± 0.59	1.22 ± 0.35	0.38 ± 0.02	0.49 ± 0.21	0.46 ± 0.14	0.30 ± 0.18	0.62 ± 0.14	0.82 ± 0.36
18:0	11.36 ± 3.80	8.92 ± 1.26	11.22 ± 1.54	9.06 ± 1.46	14.43 ± 7.45	9.77 ± 4.85	7.08 ± 0.73	7.74 ± 2.24
18:1w9	9.55 ± 3.35	18.78 ± 8.83	20.07 ± 8.26	26.32 ± 4.84	18.44 ± 16.74	16.87 ± 4.26	22.23 ± 1.57	11.11 ± 4.30
18:1w7	3.31 ± 2.19	1.69 ± 2.07	4.28 ± 4.56	0.91 ± 0.22	10.44 ± 10.28	2.30 ± 0.69	2.27 ± 2.02	3.94 ± 0.61
18:1w5	0.99 ± 0.58	0.76 ± 0.13	0.74 ± 0.34	0.57 ± 0.17	0.42 ± 0.21	0.24 ± 0.07	0.62 ± 0.02	1.99 ±1.75
18:2w6	1.34 ± 0.51	2.12 ± 1.28	2.35 ± 1.07	2.89 ± 0.77	8.12 ± 4.91	5.69 ± 3.34	9.60 ± 0.42	2.02 ± 0.54
18:4w3	1.22 ± 1.05	0.91 ± 1.57	0.76 ± 0.48	0.82 ± 0.51	0.04 ± 0.08	2.87 ± 1.06	2.24 ± 0.71	4.99 ± 2.15
20:0	1.46 ± 1.32	0.69 ± 0.21	1.18 ± 0.04	1.16 ± 0.26	1.76 ± 2.53	0.96 ± 0.79	0.60 ± 0.23	2.03 ± 2.58
20:1w11	4.50 ± 7.62	1.03 ± 0.68	0.52 ± 0.09	0.94 ± 0.61	6.02 ± 9.47	4.37 ± 4.02	2.88 ± 0.37	3.13 ± 2.35
20:1w9	3.60 ± 2.03	1.97 ± 1.08	1.35 ± 0.82	0.95 ± 0.19	2.43 ± 3.30	3.99 ± 1.29	1.03 ± 1.66	5.62 ± 3.90
20:1w7	1.18 ± 1.22	0.80 ± 0.60	0.34 ± 0.33	0.64 ± 0.33	1.85 ± 2.10	0.17 ± 0.23	0.17 ± 0.29	0.54 ± 0.48
20:3w3	1.97 ± 2.20	1.20 ± 0.67	1.65 ± 0.94	0.74 ± 0.38	0.63 ± 0.26	3.54 ± 2.76	2.36 ± 0.66	5.84 ± 1.99
20:5w3	4.84 ± 4.31	0.25 ± 0.27	1.04 ± 0.74	1.89 ± 1.31	0.33 ± 0.54	4.25 ± 3.94	1.24 ± 0.14	2.10 ± 0.93
22:0	0.91 ± 1.18	0.50 ± 0.07	0.76 ± 0.40	0.42 ± 0.03	0.45 ± 0.26	3.41 ± 2.48	0.54 ± 0.06	6.00 ± 1.83
22:1w9	3.80 ± 2.37	2.68 ± 0.52	3.14 ± 1.36	1.69 ± 1.19	1.35 ± 0.58	4.15 ± 2.43	2.51 ± 0.57	1.60 ± 1.38
22:2w6	2.23 ± 2.98	2.27 ± 3.50	0.38 ± 0.66	0.66 ± 0.75	0.66 ± 0.48	0.61 ± 0.49	0.78 ± 0.14	1.17 ± 0.38
22:5w3	2.26 ± 2.74	1.39 ± 0.81	2.03 ± 1.14	1.51 ± 0.83	0.85 ± 0.42	3.72 ± 1.25	1.91 ± 0.54	4.53 ± 1.27
22:6w3	2.60 ± 1.67	2.14 ± 2.31	2.99 ± 1.04	1.29 ± 0.39	0.97 ± 0.81	1.70 ± 1.03	1.29 ± 0.23	2.89 ± 1.10
BAME	5.12 ± 2.83	5.31 ± 0.83	3.11 ± 0.70	3.51 ± 1.87	2.30 ± 0.69	2.15 ± 1.05	4.88 ± 0.24	3.76 ± 1.84
ΣSFA	44.95 ± 3.03	47.84 ± 3.11	53.19 ± 0.40	51.13 ± 2.25	42.01 ± 7.93	38.77 ± 6.64	38.55 ± 0.44	39.61 ± 7.58
ΣMUFA	38.60 ± 6.58	41.88 ± 2.05	35.60 ± 1.37	39.07 ± 2.15	46.40 ± 5.56	38.85 ± 3.44	42.02 ± 1.55	36.85 ±2.33
ΣPUFA	16.45 ± 3.59	10.28 ± 4.95	11.21 ± 1.23	9.80 ± 2.03	11.60 ± 4.85	22.38 ± 3.53	19.43 ± 1.52	23.53 ±5.37
**B**								
	**June**				**July**			
	**1**	**2**	**3**	**4**	**1**	**2**	**3**	**4**
14:0	3.87 ± 0.89	10.50 ± 0.48	7.96 ± 2.50	6.84 ± 0.40	4.75 ± 0.89	3.34 ± 0.48	2.72 ± 1.14	2.89 ± 0.55
14:1w5	0.18 ± 0.27	0.67 ± 0.20	0.53 ± 0.31	0.72 ± 0.33	0.45 ± 0.27	0.49 ± 0.20	0.21 ± 0.14	0.50 ± 0.25
16:0	29.17 ± 5.55	31.73 ± 3.47	28.95 ± 5.26	31.35 ± 2.35	28.40 ± 5.55	14.12 ± 3.47	20.02 ± 17.55	17.17 ± 2.96
16:1w7	4.23 ± 2.00	1.43 ± 1.15	2.44 ± 1.95	6.44 ± 6.65	3.99 ± 2.00	3.09 ± 1.15	8.25 ± 9.43	1.56 ± 0.39
16:1w5	1.51 ± 2.58	1.93 ± 0.47	2.03 ± 2.20	1.35 ± 1.17	5.45 ± 2.58	5.36 ± 0.47	4.05 ± 0.87	4.19 ± 0.65
17:1w7	0.28 ± 0.04	0.31 ± 0.09	0.44 ± 0.39	0.96 ± 1.17	0.15 ± 0.04	0.25 ± 0.09	0.29 ± 0.16	0.31 ± 0.32
18:0	5.98 ± 3.15	12.42 ± 1.51	10.68 ± 3.96	14.76 ± 7.56	12.50 ± 3.15	8.19 ± 1.51	15.01 ± 5.78	7.30 ± 4.11
18:1w9	18.13 ± 2.50	12.26 ± 1.97	10.28 ± 10.11	2.82 ± 2.67	12.95 ± 2.50	9.66 ± 1.97	10.20 ± 3.03	12.24 ± 4.18
18:1w7	3.08 ± 1.47	3.15 ± 0.22	5.22 ± 2.63	2.52 ± 1.68	5.29 ± 1.47	4.31 ± 0.22	4.36 ± 0.12	9.56 ± 8.02
18:1w5	1.78 ±1.75	0.07 ± 1.65	1.66 ± 1.01	0.57 ± 0.75	3.61 ± 1.75	3.79 ± 1.65	3.42 ± 1.12	2.78 ± 1.90
18:2w6	3.26 ± 1.01	2.25 ± 2.76	1.73 ± 1.47	0.84 ± 0.69	3.74 ± 1.01	9.63 ± 2.76	4.40 ± 3.62	22.06 ± 2.83
18:4w3	3.61 ± 2.75	0.77 ± 0.67	0.53 ± 0.68	2.22 ± 0.75	2.17 ± 2.75	1.99 ± 0.67	2.41 ± 1.84	1.08 ± 0.64
20:0	7.25 ± 1.55	1.32 ± 3.18	1.82 ± 1.26	1.75 ± 0.38	1.87 ± 1.55	4.43 ± 3.18	1.25 ± 0.16	2.98 ± 2.10
20:1w11	3.21 ± 0.04	0.69 ± 1.40	1.87 ± 0.44	0.79 ± 0.23	0.52 ± 0.04	3.08 ± 1.40	3.01 ± 2.32	1.23 ± 0.54
20:1w9	1.55 ± 0.24	0.85 ± 2.27	1.30 ± 0.85	1.20 ± 1.29	0.37 ± 0.24	4.14 ± 2.27	1.77 ± 0.53	0.79 ± 0.09
20:1w7	0.38 ± 0.43	0.22 ± 1.36	0.60 ±0.22	1.53 ± 1.86	0.39 ± 0.43	3.07 ± 1.36	0.63 ± 0.62	0.69 ± 0.67
20:3w3	0.57 ± 0.49	0.95 ± 0.42	0.87 ± 0.78	0.95 ± 1.09	0.66 ± 0.49	0.67 ± 0.42	0.75 ± 0.32	0.33 ± 0.13
20:5w3	1.54 ±0.56	1.40 ± 2.49	1.60 ± 0.57	1.33 ± 1.01	1.03 ± 0.56	3.48 ± 2.49	1.66 ± 0.77	1.49 ± 0.45
22:0	1.38 ± 0.10	2.12 ± 0.01	3.00 ± 1.96	1.00 ± 0.14	0.67 ± 0.10	0.48 ± 0.01	0.42 ± 0.15	1.25 ± 1.22
22:1w9	1.96 ± 2.04	4.00 ± 2.56	2.86 ± 1.63	2.27 ± 3.28	2.40 ± 2.04	4.54 ± 2.56	2.92 ± 1.75	2.04 ± 0.73
22:2w6	0.26 ± 0.11	1.14 ± 0.27	1.21 ± 0.67	1.86 ± 2.19	0.37 ± 0.11	1.02 ± 0.27	0.41 ± 0.11	0.29 ± 0.30
22:5w3	0.95 ± 0.47	1.25 ± 1.30	2.92 ± 1.42	2.14 ± 1.83	1.30 ± 0.47	1.52 ± 1.30	0.71 ± 0.29	0.49 ± 0.20
22:6w3	1.96 ± 0.57	2.48 ± 1.66	3.34 ± 2.30	2.64 ± 2.24	1.19 ± 0.57	2.62 ± 1.66	2.03 ± 0.74	1.19 ± 0.24
BAME	3.90 ± 0.68	6.10 ± 2.29	6.16 ± 2.56	11.14 ± 7.16	5.78 ± 0.68	6.72 ± 2.29	9.09 ± 5.60	5.60 ± 1.54
ΣSFA	51.56 ± 6.17	64.20 ± 6.90	58.56 ± 8.55	66.85 ± ##	53.98 ± 6.17	37.29 ± 6.90	48.52 ± 16.53	37.19 ± 7.68
ΣMUFA	36.29 ± 9.30	25.56 ± 6.11	29.25 ± ###	21.16 ± ##	35.56 ± 9.30	41.79 ± 6.11	39.11 ± 13.89	35.89 ± 5.05
ΣPUFA	12.15 ± 3.51	10.24 ± 8.31	12.19 ± 4.93	11.99 ± 9.07	10.46 ± 3.51	20.92 ± 8.31	12.37 ± 3.10	26.92 ±2.77

Only FA > 1% are displayed. PUFA = Polyunsaturated Fatty Acids, MUFA = Monounsaturated Fatty Acids, SFA = Saturated Fatty Acids, BAME = Bacterial Fatty Acids.

#### Consumers

A total of 28 and 29 FA contributing > 1% to TFA were found in the adductor muscles and the gonads respectively, but their compositions differed (Tables B and C in S1 File). The major FA found in adductor muscles were 16:0 (18–23%), 18:0 (5–7%), 22:2 Non-methylene-interrupted FA (NMI; 4–5%), 20:2NMI1 (5–7%), 20:5w3 (10–13%) and 22:6w3 (12–20%); while those in gonad tissues were 16:0 (20–23%), 16:1w7 (3–7%), 18:0 (3–7%), 20:5w3 (12–27%) and 22:6w3 (10–26%).

The main source of difference amongst all samples was between the two types of tissues (PERMANOVA, *p* < 0.001). SIMPER highlighted that the main differences between the tissues were due to higher proportions of 18:1w9, diatom trophic markers (TM, 16:1w7 and 20:5w3) and dinoflagellate TM (18:4w3 and 22:6w3) in gonads; and higher proportions of 18:0, 20:0, 20:2NMI1, 20:4w6 and 22:2NMI1 in the adductor muscles, explaining 55% of the FA differences between the two tissues (S2 Fig). Gonads also showed a higher proportion of essential FA (EFA; 46% of TFA) than muscles (26–35% of TFA) at all sites and across all months. In particular, gonads had twice the proportion of 20:5w3 compared to the adductor muscles. Considering the strong dissimilarities between gonads and adductor muscles, the FA compositions of these tissues were investigated separately in the remaining analyses.

#### Consumers: adductor muscle

Adductor muscle samples were characterized by high proportions of PUFA throughout the year (48–57%), followed by SFA (31–42%) and MUFA (10–16%; Table 2). PERMANOVA showed that in December the factor site was significant (Table 3), indicating that the FA signatures of the mussels differed across sites. Similarly, during the month of February, the only source of variation among muscle samples was the factor site, with no significant effect of the factor upwelling (Table 3).

**Table 2:** Multiple mean comparisons of the main fatty acid trophic markers of the adductor muscle and gonad of *Mytilus galloprovincialis* among the four sites in a) December and February and b) June and July. The values for ΣSFA, ΣMUFA, ΣPUFA and ΣEFA are percentages expressed as mean ± standard deviation (n = 6 per site).

A								
	**December**				**February**			
	**1**	**2**	**3**	**4**	**1**	**2**	**3**	**4**
**Adductor muscle**								
ΣSFA	34.53+1.06	35.02+0.67	34.07+1.54	34.78+0.97	33.00+1.20	33.29+0.32	31.44+2.19	31.67+1.29
ΣMUFA	10.88+0.97	10.15+0.72	11.85+2.31	11.76+1.14	16.33+2.59	15.17+2.19	12.76+2.42	10.82+2.34
ΣPUFA	54.59+1.24	54.83+0.86	54.07+2.63	53.46+1.18	50.67+2.22	51.54+2.11	55.81+3.84	57.52+3.51
ΣEFA	32.91+0.66	33.47+1.25	36.76+2.60	33.80+2.69	30.08+2.16	29.20+3.03	33.67+2.34	26.61+1.53
20:5w3/22:6w3	0.73	0.85	0.59	0.66	1.02	1.22	0.63	0.73
**Gonad**								
ΣSFA	34.82+3.15	31.00+2.48	34.54+2.76	31.78+2.00	31.61+2.54	33.58+1.67	33.76+5.40	31.76+1.24
ΣMUFA	13.66+4.63	15.14+2.76	11.02+4.96	17.72+4.82	18.40+4.69	15.46+4.18	12.13+5.02	17.97+3.74
ΣPUFA	51.52+3.15	53.86+2.36	54.44+4.93	50.51+4.14	49.99+3.19	50.96+3.74	54.11+1.85	50.27+3.70
ΣEFA	40.11+5.12	40.96+2.91	45.07+8.36	36.95+4.52	37.65+4.06	39.54+4.95	41.30+4.74	36.39+3.38
20:5w3/22:6w3	1.77	1.74	0.58	1.23	1.73	2.84	0.66	1.49
B								
	**June**				**July**			
	**1**	**2**	**3**	**4**	**1**	**2**	**3**	**4**
**Adductor muscle**								
ΣSFA	35.80+2.26	34.82+1.21	35.72+2.49	37.40+3.00	41.42+6.98	35.16+1.48	37.22+4.28	38.00+4.46
ΣMUFA	11.98+1.58	11.77+1.29	12.24+2.31	10.63+0.93	10.60+0.52	11.66+2.18	11.52+2.44	11.84+1.97
ΣPUFA	52.23+1.74	53.41+0.76	52.04+1.26	51.97+3.59	47.98+6.85	53.18+2.49	51.26+2.88	50.16+3.63
ΣEFA	33.31+3.01	35.89+2.37	35.91+1.39	33.77+2.84	28.34+7.87	35.94+4.13	35.89+3.99	31.60+3.43
20:5w3/22:6w3	0.80	1.26	0.61	0.49	0.76	1.15	0.61	0.52
**Gonad**								
ΣSFA	30.96+1.90	32.18+1.18	33.04+2.12	33.02+0.65	31.16+7.09	34.40+2.70	34.11+1.67	32.67+1.38
ΣMUFA	14.39+3.69	14.64+2.64	16.37+3.84	12.66+3.14	13.51+2.68	12.24+3.82	10.94+3.24	13.09+3.39
ΣPUFA	54.65+2.94	53.18+2.28	50.58+4.18	54.32+3.19	55.33+4.55	53.37+1.48	54.95+1.67	54.23+2.75
ΣEFA	42.43+4.32	40.16+1.95	39.48+4.78	41.63+4.33	40.41+2.60	41.66+3.13	46.56+4.36	40.49+4.79
20:5w3/22:6w3	1.27	1.68	0.61	0.49	1.12	1.48	0.68	0.57

Only FA > 1% were used for the analyses. PUFA = Polyunsaturated Fatty Acids, MUFA = Monounsaturated Fatty Acids, SFA = Saturated Fatty Acids, EFA = Essential Fatty Acids (20:4w6, 20:5w3 and 22:6w3).

**Table 3:** PERMANOVA results on the fatty acid composition of the adductor muscles of *Mytilus galloprovincialis* at four sites and across four month on the South African west coast.

December						February						June- July					
	df	MS	Pseudo-F	*p*			df	MS	Pseudo-F	*p*			df	MS	Pseudo-F	*p*	
Si	3	74.09	4.40	0.000	***	Up	1	173.65	1.01	0.669		Month	1	40.08	1.23	0.282	
Res	20	16.84				Si (Up)	2	171.47	7.01	0.000	***	Si	3	268.35	8.24	0.000	***
						Res	20	24.454				Month x Si	3	33.21	1.02	0.439	
												Res	40	32.58			

Up = Upwelling, Si = Site, df = degrees of freedom, MS = mean square;

* *p* < 0.05;

** *p* < 0.01;

*** *p* < 0.001.

In December, sites 1 and 2 were different from each other and from all the other sites (PERMANOVA post hoc pair-wise test, p < 0.05) by being characterized by SFA, 20:4w6 and 22:2w6 in the case of site 1, while site 2 had high percentages of diatom TM and 22:2NMI1. Sites 3 and 4 did not differ from each other and they exhibited a high proportion of 18:1w9, dinoflagellate TM and 20:1w11. These FA contributed to 55% of the dissimilarity among sites (SIMPER).

In February, only the two non-upwelling sites, 2 and 4, were significantly different from each other, while sites 1 and 3 did not differ (PERMANOVA *post hoc* pair-wise test and PCA). SIMPER showed that sites 1, 2 and 3 had higher proportions of 14:0 and diatom TM than site 4 (20% of TFA), which was enriched in, 20:2NMI1, 22:2w6 and 20:4w3 (17% of TFA; Table B in S1 File). This is partially in agreement with the ratio 20:5w3/ 22:6w3, which discriminates between diatoms (> 1) and dinoflagellates (< 1) ([50,51]) and indicated a predominance of diatoms in mussel diets (values > 1) in February at sites 1 and 2.

**Fig 3:**
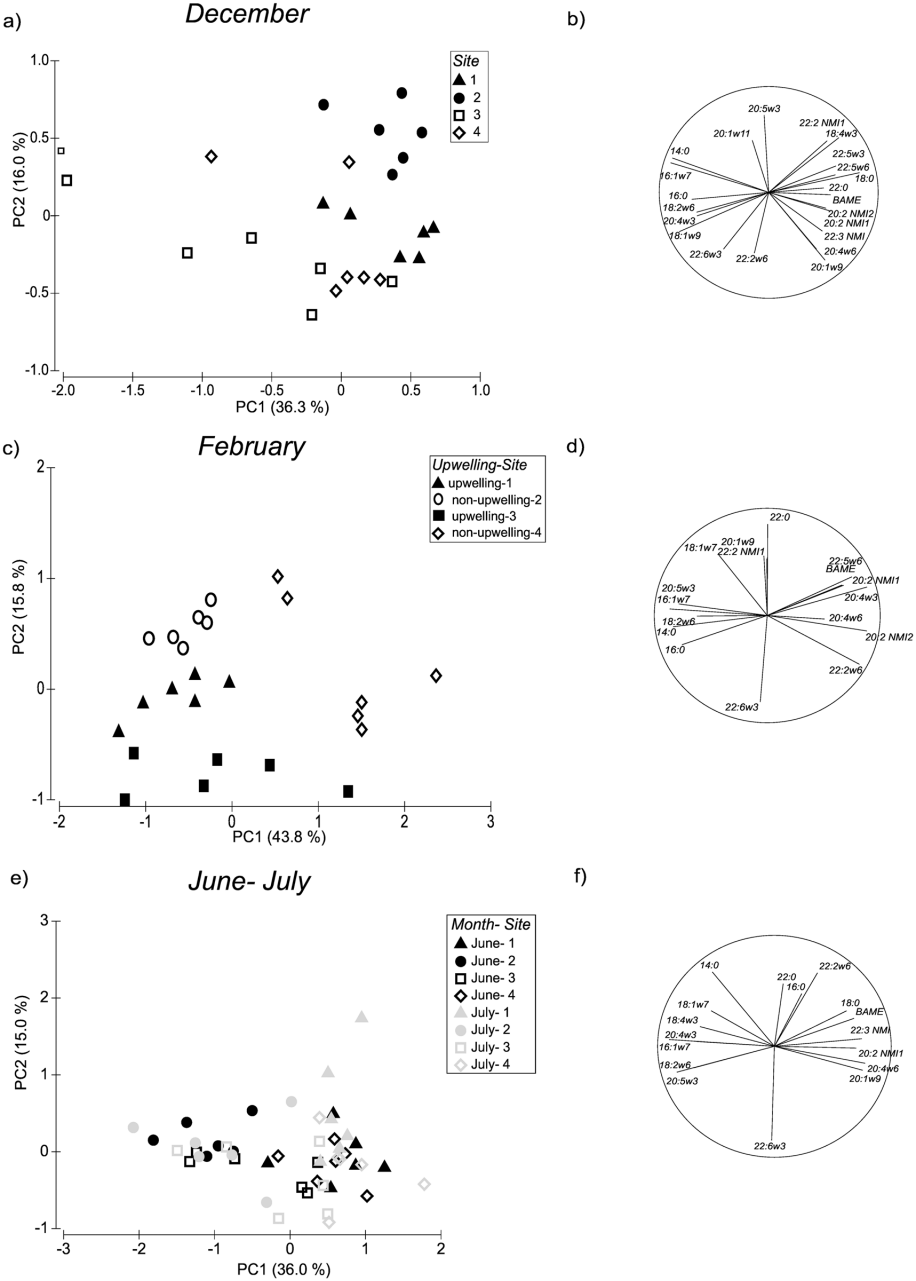
PCA conducted on the adductor muscles of *Mytilus galloprovincialis* collected at rocky shore sites, on the South African west coast in a) December, c) February e) June and July. Each symbol represents a single replicate. b, d, f) Eigenvalues for each of the factors (fatty acids). The circle corresponds to eigenvalues of −1 to 1. For clarity, only fatty acids with eigenvalues > 0.5 are shown.

For June and July, PERMANOVA highlighted a significant effect of the factor site only (Table 3). All sites were different from each other in both months, with the exception of sites 1 and 4 which did not differ. As for December, specimens at site 2 were typified by diatom TM, site 3 by dinoflagellate TM, while sites 1 and 4 were characterized by 18:1w9, 20:1w9, 20:2NMI1, 20:4w6 and 22:2w6 (SIMPER, Fig 3). The ratio 20:5w3/ 22:6w3 again showed a dominance of dinoflagellates at all sites with the exception of site 2 in June and July when it was diatom dominated.

#### Consumers: gonad

As for the adductor muscle, gonad FA compositions included a high proportion of PUFA at all sites and months (50–55% of TFA), followed by SFA (30–35%) and MUFA (10–15%; Table 2). The 20:5w3/22:6w3 ratio was variable among sites: at sites 1 and 2 the ratio was > 1 across all months; at site 3 it was < 1, while at site 4 it was > 1 during the summer months and decreased to < 1 in the winter period. PERMANOVA showed that the only significant factor for the analyses performed was site, with no effect of upwelling in February, indicating that patterns in the FA composition of gonads differed across sites and that these differences remained constant among months (Table 4). Axes 2 of the PCA showed a partial separation between samples of sites 1–2 and sites 3–4 in December, February and June- July (S3 Fig). This pattern was not confirmed by the PERMANOVA pairwise test, however, which showed that only sites 3 and 4 did not differ from each other in all months (*p* > 0.05). Thus sites were different among each other, however, we could not identify a clear pattern of separation (S3 Fig).

**Table 4:** PERMANOVA results on the fatty acid compositions of gonads of *Mytilus galloprovincialis* at four sites and across four month on the South African west coast in relation to the different factors.

December						February						June- July					
	df	MS	Pseudo-F	*p*			df	MS	Pseudo-F	*P*			df	MS	Pseudo-F	*p*	
Si	3	267.79	2.96	0.004	**	Up	1	188.08	0.82	1		Month	1	121.41	2.32	0.070	
Res	20	90.479				Si (Up)	2	228.69	4.53	0.004	**	Si	3	355.57	6.79	0.000	***
						Res	20	50.512				Month x Si	3	64.39	1.23	0.273	
												Res	38	52.34			

Up = Upwelling, Si = Site, df = degrees of freedom, MS = mean square;

* *p* < 0.05;

** *p* < 0.01;

*** *p* < 0.001.

### Indices

#### Condition Index

**Fig 4:**
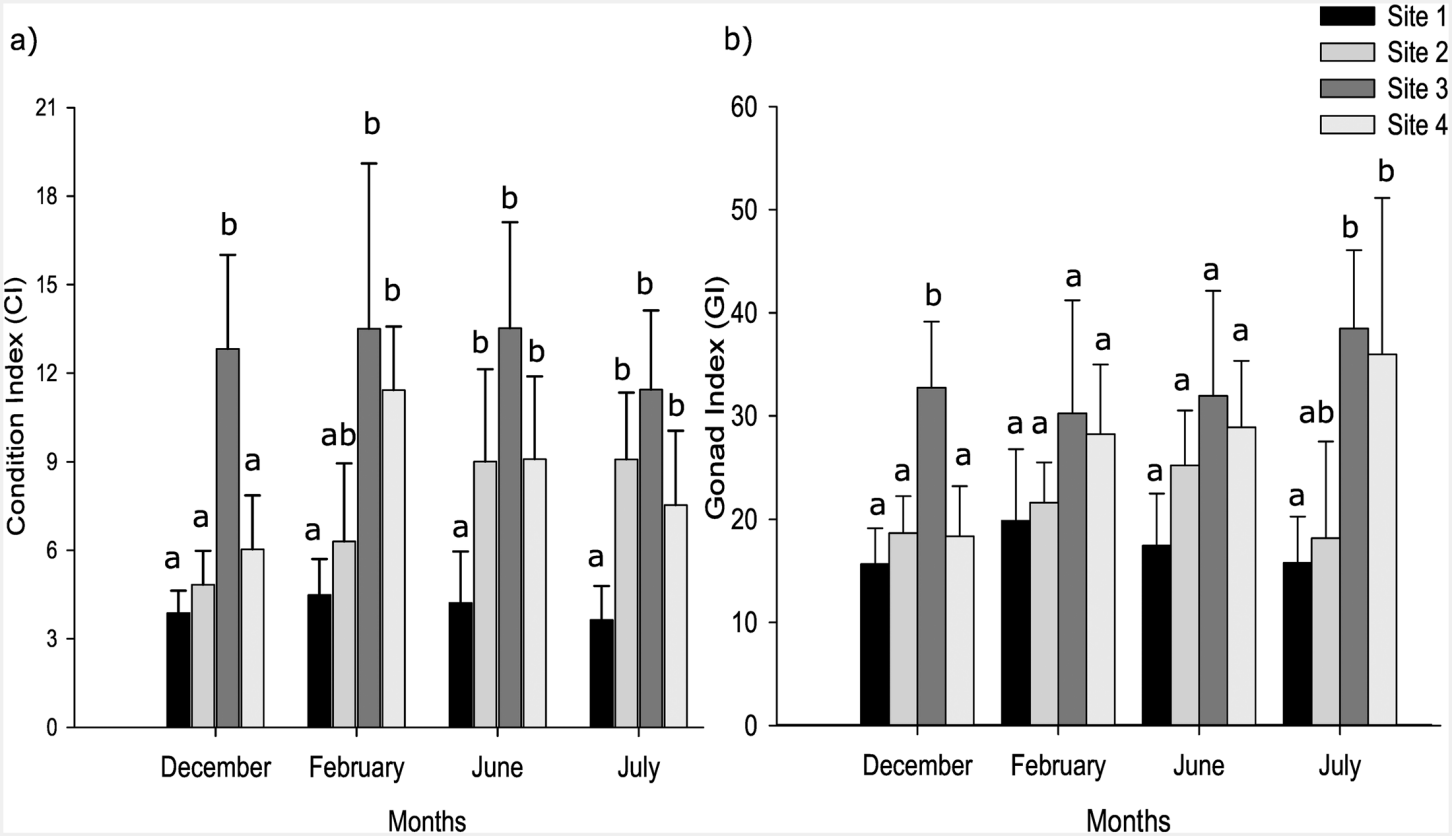
a) Condition Index and b) Gonad Index of *Mytilus galloprovincialis* at the four sites and across the four months sampled (n = 26 and n = 6 for CI and GI, respectively). Sites are arranged from south to north (left to right). Values are means, the error bars indicate standard deviations. Letters indicate homogenous groups within months (*p* > 0.05).

Month did not affect CI in winter, nor did upwelling have a significant effect in February (ANOVA, *p* > 0.05 for both analyses), however, there were significant effects of the factor site in all analyses (ANOVA, *p* < 0.001) and the interaction between site and month in June-July (ANOVA, *p* < 0.05). Tukey HSD tests showed that site 1 had the lowest CI (4.05 ± 1.20; *p* < 0.001, Fig 4a), while site 2 had CI values that were intermediate between those of site 1 and sites 3–4. These latter sites were statistically different only in December, when CI values at site 3 were significantly higher than at the other three sites (12.80 ± 3.20; *p* < 0.01). In addition, CI at site 2 increased from the summer to the winter months (5.55 ± 1.9–9.05 ± 2.70). The results indicate a clear south-north gradient of increasing CI from sites 1 to 3, followed by a decrease at site 4, a pattern that was consistent across all months.

#### Gonad Index

The three analyses indicated that the only significant effects were of site and its interaction with month in the winter months (ANOVA, *p* < 0.01 for all analyses), with no upwelling effect in February. In December, GI at site 3 was significantly higher than the other three sites (Tukey HSD, *p* < 0.05), which did not differ from each other. In February and June the four sites did not differ (Tukey HSD, *p* > 0,05), while in July sites 3 and 4 had higher GI values than sites 1, and site 2 was not different from the other sites (Tukey HSD, *p* < 0.01; Fig 4b). Although differences among sites were not always significant, the spatial pattern was identical to that for CI, with a trend of increasing values from site 1 to site 3 followed by a slight decrease at site 4.

## Discussion

Using FA techniques, we aimed to assess the effects of upwelling on the dietary signatures of benthic filter feeders in the Benguela upwelling region, by comparing sites that are considered to be strong upwelling centres with downstream sites, which may be influenced only indirectly by upwelling.

The temperature loggers confirmed the categorisation of sites 1 and 3 as upwelling centres [37], but with upwelling occurring only during summer, predominantly in January and February, as previously shown [34,19] and *in situ* temperatures indicated non-upwelling conditions at all sites during the winter sampling months from April to July. Throughout summer, from December to March, the upwelling sites were colder and were characterized by frequent, intense upwelling events, with drops in mean daily temperatures of as much as 8°C. Site 2 clearly behaved as a non-upwelling site, while site 4 had temperatures that were on average higher than at the upwelling sites, but it did experience two weak upwelling events during the study. This reflects the inter-annual variability in upwelling frequency and intensity that has been shown for the Benguela and other upwelling systems [52,53].

We anticipated a stronger diatom FA signature at upwelling sites than at downstream sites, and expected the difference to be even more pronounced during the upwelling season. The SPM data did not support this hypothesis, with no differences in the SPM FA composition among sites or sampling events. SPM is an instantaneous measure, however, reflecting the FA composition of the food available for filter feeders at the moment of the sampling, while the FA turn-over rate in mussels is approximately one month [35]. Consequently, the SPM collected on the same day as the mussels does not necessary represent what the organisms were feeding on a few days/weeks before. We did not, therefore, expect a strong link between the FA composition of the SPM and the mussels, but we did expect some differences in the SPM signatures in relation to upwelling. The spatial and temporal homogeneity of the SPM even when temperature data indicated the occurrence of upwelling, was unexpected and suggests either that upwelling influenced SPM conditions in a homogeneous way across the study region, or that changes in primary producers were too rapid to be detected with our approach.

Although there was a predominance of phytoplankton in the diets of our specimens, the results for adductor muscle indicated idiosyncrasy among sites, with no significant effect of upwelling. Specimens from site 3 were characterized by dinoflagellate TM throughout the investigation, and the ratio of 20:5w3/22:6w3 indicated a generally higher contribution of dinoflagellates than diatoms (ratio < 1 [50,51]) at sites 1, 3 and 4, while the presence of diatom TM in all months and a ratio of > 1 in all sampling events indicated a predominance of diatoms in the diets of mussels at site 2. The presence of the cold, nutrient-rich Benguela Current on the South African west coast promotes high primary production dominated by phytoplankton [32,54,55], explaining the strong phytoplankton FA signatures at all sites observed for *M*. *galloprovincialis*. Gonad has rapid turnover rates, so that its FA signatures change more rapidly than those of the more conservative adductor muscle [56]. Gonad showed marked variability in the 20:5w3/22:6w3 ratio and in the FA composition among sites and months, but in this case, there was no clear pattern.

Although both CI and GI showed strong variability among sites, with no significant effects of the factors upwelling or month, both exhibited a marked geographic gradient. In all months, values for both indices increased from south to north, from site 1 to site 3, slightly dropping at site 4, with site 1 showing the lowest CI across all months and site 3 the highest. This pattern is intriguing as temperature data indicated strong upwelling at site 1 during summer, and we expect higher phytoplankton concentrations during upwelling events [7,57]. The low CI suggests, however, that the conditions at this site were less favourable for *M*. *galloprovincialis* than at the other sites. The low water temperatures experienced by animals at site 1 are unlikely to be responsible for the low CI measured. The average seawater temperature for the study period was approximately 13–15°C with minimum values down to 9–10°C. Some studies have shown that temperatures < 13°C are suboptimal for *M*. *galloprovincialis* [58,59], but another showed that the thermal limits of this species were between 5–27°C [60]. *M*. *galloprovincialis* is well represented in the intertidal zone of the west coast of South Africa; it has displaced the indigenous species to become the dominant mussel along this coast [61] and is well-adapted to cold environments [62,60]. More plausibly, the results may reflect low food availability at this site as has been observed in other systems [63,64]. Field et al [65] conducted daily measurements for 30 days at a site just 2 km from our site 1 and showed low nearshore chlorophyll *a* concentrations during upwelling driven by strong offshore winds. Consequently, phytoplankton blooms supported by this nutrient injection develop over the course of several days farther offshore and are advected to downstream areas. In contrast, the non-upwelling site 4 had CI values comparable to site 3, which had the highest CI on three of the four sampling events. This presumably reflects topographic effects, as site 4, Elandsbaai, is located in a bay, where chlorophyll *a* and mussel growth are likely to be greater due to retention of particles [66]. CI thus seems to reflect not just the effects of upwelling on primary production, but the effects of local hydrodynamics which determine whether enhanced productivity is retained near or advected away from sites.

GI showed an identical pattern to CI, but values at site 1 were comparable to the other sites. This suggests that animals at site 1 invested as heavily in reproduction as the other populations despite experiencing unfavourable conditions and low CI values. Investment in reproduction has been shown to be independent of body condition and feeding regime in other invertebrates such as clams [67] and squid [68]. Importantly the GI in mussels is closely related to the reproductive phase: the GI will be low if an individual has just spawned and high if it is collected immediately before spawning. According to van Erkom Schurink & Griffiths [69], *M*. *galloprovincialis* in South Africa has a peak spawning period in summer (November- December) and one in winter (April- June), though the timing of mussel spawning can change markedly [70]. Although we have no information on the reproductive state of the sampled populations or whether they were reproductively synchronised, the results were consistent across the months sampled, indicating consistency in the pattern observed.

The clearest results highlighted strong differences in the FA compositions of gonad and the more stable adductor muscle tissues, with gonad showing a higher proportion of EFA (*i*.*e*. 20:4n-6, 20:5n-3 and 22:6n-3; [71]) across all months and sites. EFA are critical for the maintenance of cell membrane structure and function. They are also precursors of bioactive compounds that are essential for survival, growth and reproduction [72,73], however they cannot be synthetized *de novo* in sufficient quantities and must be acquired through the diet [74,75]. Previous studies have suggested preferential retention of specific FA in the gonad, in order to ensure reproduction and to improve offspring survival [76–78]. Increased concentrations of specific FA in the gonads during reproduction have been shown for a range of animals including octopuses [79], fishes [80] and bivalves [81]. *In situ* temperature loggers used to identify upwelling conditions at the four sites indicated that such events occurred only at the upwelling centres and only in austral summer. The high proportion of EFA we found, especially of 20:5w3, suggests preferential retention of EFA in the gonads, presumably for reproductive purposes. The contrast between GI and CI results supports this interpretation. Although both GI and CI were consistently low at site 1, the differences were only significant for CI, suggesting that investment of resources into reproduction was a priority, maintained even at the cost of adult condition. The strong dissimilarities between gonad and muscle may also reflect the very different turnover rates of the two tissue types, with gonad changing FA signatures faster than adductor muscle [56,82]. Upwelling or cooling events occurred in late December-February and adductor muscle samples from February were characterized by a high proportion of diatom TM. This matches the one month FA turn-over period found for mussels [35], reflecting the effects of upwelling from the previous month. However, this effect was not detectable in gonads, which showed differences in FA signatures, but without a clear pattern.

We found no direct effect of upwelling on the FA composition of muscle or gonad tissues. However, strong dissimilarities were found among sites which were mostly consistent across the four months sampled. Adductor muscle at non-upwelling site 2 were characterised by diatom TM, at the upwelling site 3 they were enriched in dinoflagellate TM, while at sites 1 and 4 muscle samples generally had high proportions of PUFA, though no specific FATM were identified. These results partially contradict our expectations of stronger phytoplankton TM at upwelling centres, with the two upwelling sites behaving in opposite ways. Site 3 conformed to the general conception of a late stage of upwelling on three of the four sampling events, being characterised by dinoflagellate TM (post diatom blooms), but site 1 was not typified by any distinct phytoplankton TM. Likewise, site 2, which is supposedly a downstream, non-upwelling site, had a strong diatom signature. Similar disparities were found with the condition indices. Site 3 had the highest CI and GI among all sites and in all months, while site 1 showed the lowest. However, *in situ* temperature data indicated that, during the upwelling season, both upwelling sites were exposed to long periods of upwelled water, while the two non-upwelling sites, with two exceptions at site 4, experienced cooling events that were less frequent, less protracted and less intense. The effects of upwelling can be interpreted in two ways. At upwelling sites, nutrients and particles can be upwelled close to the coast and then moved offshore with the surface water, resulting in phytoplankton-poor waters inshore [65,83–85]. Offshore incubation of these enriched waters can be followed by onshore advection (either to the same point on the shore or farther downstream) during upwelling relaxation or downwelling. Thus, the retention time of the newly upwelled water can be too short to allow bloom development at the upwelling site itself, while phytoplankton blooms are detectable downstream of the upwelling centre, so that downstream sites can exhibit higher phytoplankton concentrations than the upwelling centres themselves. Other studies have indicated that upwelling stimulates phytoplankton and macrophyte growth at the site of upwelling, which suggests a very localised effect [86,1]. In our study, the first scenario seems to apply to the comparison of sites 1 and 2, and the second to sites 3 and 4, presumably reflecting differences in nearshore hydrodynamics in the two regions. For instance, local hydrodynamics may result in offshore advection of water at site 1 but retention at site 3. Although we could not classify sites as either upwelling or non-upwelling in the month of December, the FA results conformed to expectations based on such categorisation, showing dinoflagellate TM at site 3 and diatom TM at site 2, as a site downstream of site 1. This agrees with the fact that upwelling occurs during the summer months in the Southern Benguela area and that the sites we chose *a priori* as upwelling or non-upwelling sites for the comparison conformed to this classification. Our results partially contradict the findings of Xavier et al [37]. Upwelling has long been identified as a key process influencing the advection of planktonic larvae to/from the nearshore [87,88] and, using the same sites as us, Xavier et al [37] found higher recruitment of the mussel *M*. *galloprovincialis* at upwelling centres than at the downstream sites. The discrepancy between the two investigations may reflect inter-annual variability in upwelling intensity and frequency as is known to occur in this area [52,53], or may more simply indicate that nutrient levels, phytoplankton response and the advection of mussel recruits all respond to upwelling with very different lag periods. Another important aspect to highlight is that the differences observed among sites were consistent in both the upwelling and non-upwelling seasons, suggesting that differences in the conditions at these sites perpetuate across the upwelling and non-upwelling seasons.

Adductor muscle tissue had a strong phytoplankton FA signature at all sites in February, during the upwelling season, suggesting that a very strong upwelling event occurred during the previous weeks, as was shown by the temperature data. However, even if upwelled waters were present only during the summer months, the tissues of mussels showed similar proportions of PUFA-MUFA-SFA over time, indicating that the specimens were exposed to similar food in both austral summer and winter and under both upwelling and non-upwelling conditions. The fact that the factor upwelling was not significant in any of the analyses, suggests that the entire study area is affected by upwelling, indicating that the influence of upwelling in the southern Benguela system is pervasive, rather than discrete. This partially contrasts with a similar study conducted over a larger scale with more widely separated sites on the same coast [89]. On comparing sites spread across larger scales (approximately 400 km of the west coast of South Africa), the effects of upwelling were found to be discrete, with mussels and barnacles at upwelling sites having different FA and stable isotope signatures from specimens at non-upwelling sites in the same biogeographic province [89]. The discrepancy between the two studies presumably reflects their different spatial resolutions, as well as their geography. One study was conducted over 400 km of coastline, including an area close to the Luderitz upwelling cell in Namibia, which is characterised by frequent and intense upwelling events throughout the year, whereas the other encompassed 200 km of coast where upwelling occurs only seasonally [19,32,90]. Thus, depending on the spatial scale of resolution and the nature of upwelling, its effects can be either discrete or diffuse. We tend to characterise upwelling as a binary phenomenon, but clearly, the intensity and frequency of upwelling events are critical, as are the effects of nearshore hydrodynamics, which can either retain nutrient enriched waters and the associated primary production locally or export them to downstream areas.

Overall, we demonstrated upwelling at sites categorised *a priori* as upwelling centres, but only during the upwelling season. Despite the seasonal differences in upwelling conditions at these sites compared to the non-upwelling sites, we found no clear differences in the FA of mussels at the two types of sites, indicating that the effects of nutrient enrichment were diffuse across the study area. Critically, we found that upwelling centres behaved differently, presumably reflecting the nature of retention and advection of upwelled water.

## Supporting Information

S1 Fig a) PCA conducted on the fatty acid composition of suspended organic matter (SPM) collected from intertidal rocky shores at four sites during the four sampling events. Each symbol represents a single replicate. Upwelling did not have an effect on the fatty acid signatures of samples from February and thus we show a single PCA with the samples of all months together. b) Eigenvalues for each of the factors (fatty acids). The circle corresponds to eigenvalues of −1 to 1. For clarity, only fatty acids with eigenvalues > 0.5 are shown.
(TIF)

S2 Fig a) PCA conducted on the adductor muscle and gonad tissues of *Mytilus galloprovincialis* collected at intertidal rocky shores at each site during the four sampling events. Each symbol represents a single replicate. b) Eigenvalues of each of the factors (fatty acids). The circle corresponds to eigenvalues of −1 to 1. For clarity, only fatty acids with eigenvalues > 0.5 are shown.
(TIF)

S3 Fig PCA conducted on the fatty acid composition of gonad tissue of *Mytilus galloprovincialis* collected from intertidal rocky shores at four sites during a) December c) February e) June and July. Each symbol represents a single replicate. b), d) and f) Eigenvalues of each of the factors (fatty acids). The circle corresponds to eigenvalues of −1 to 1. For clarity, only fatty acids with eigenvalues > 0.5 are shown.
(TIF)

S1 File Supplementary material. (DOCX)

## References

[1] Wieters EA. Upwelling control of positive interactions over mesoscales: a new link between bottom-up and top-down processes on rocky shores. Mar Ecol Prog Ser. 2005;301: 43–54.

[2] Blanchette CA, Broitman BR, Gaines SD. Intertidal community structure and oceanographic patterns around Santa Cruz Island, CA, USA. Mar Biol. 2006;149: 689–701. 10.1007/s00227-005-0239-3

[3] Menge BA, Daley BA, Wheeler PA, Dahlhoff E, Sanford E, Strub PT. Benthic–pelagic links and rocky intertidal communities: bottom-up effects on top-down control? Proc Natl Acad Sci. 1997;94: 14530–14535. 9405647 10.1073/pnas.94.26.14530 PMC25044

[4] McQuaid CD, Payne AIL. Regionalism in marine biology: the convergence of ecology, economics and politics in South Africa. South Afr J Sci. 1998;94: 433–436.

[5] Connell JH. The consequences of variation in initial settlement vs. post-settlement mortality in rocky intertidal communities. J Exp Mar Biol Ecol. 1985;93: 11–45. 10.1016/0022-0981(85)90146-7

[6] Raimondi PT. Patterns, mechanisms, consequences of variability in settlement and recruitment of an intertidal barnacle. Ecol Monogr. 1990;60: 283–309. 10.2307/1943059

[7] Figueiras FG, Labarta U, Reiriz MJF. Coastal upwelling, primary production and mussel growth in the Rías Baixas of Galicia In: Vadstein O, Olsen Y, editors. Sustainable Increase of Marine Harvesting: Fundamental Mechanisms and New Concepts. Springer Netherlands; 2002 pp. 121–131. Available: http://link.springer.com/chapter/10.1007/978-94-017-3190-4_11

[8] Bosman AL, Hockey P a. R, Siegfried WR. The influence of coastal upwelling on the functional structure of rocky intertidal communities. Oecologia. 1987;72: 226–232. 10.1007/BF00379273 28311545

[9] Nielsen KJ. Nutrient loading and consumers: agents of change in open-coast macrophyte assemblages. Proc Natl Acad Sci. 2003;100: 7660–7665. 10.1073/pnas.0932534100 12796509 PMC164644

[10] Smith JR, Fong P, Ambrose RF. Spatial patterns in recruitment and growth of the mussel *Mytilus californianus* (Conrad) in southern and northern California, USA, two regions with differing oceanographic conditions. J Sea Res. 2009;61: 165–173. 10.1016/j.seares.2008.10.009

[11] Cury P, Roy C. Optimal environmental window and pelagic fish recruitment success in upwelling areas. Can J Fish Aquat Sci. 1989;46: 670–680. 10.1139/f89-086

[12] Ryther JH. Photosynthesis and fish production in the sea. Science. 1969;166: 72–76. 10.1126/science.166.3901.72 5817762

[13] Bustamante RH, Branch GM, Eekhout S, Robertson B, Zoutendyk P, Schleyer M, et al Gradients of intertidal primary productivity around the coast of South Africa and their relationships with consumer biomass. Oecologia. 1995;102: 189–201. 10.1007/BF00333251 28306874

[14] Sanford E, Menge BA. Spatial and temporal variation in barnacle growth in a coastal upwelling system. Mar Ecol Prog Ser. 2001;209: 143–157.

[15] Ventura CRR, Falcao APC, Santos JS, Fiori CS. Reproductive cycle and feeding periodicity in the starfish *Astropecten brasiliensis* in the Cabo Frio upwelling ecosystem (Brazil). Invertebr Reprod Dev. 1997;31: 135–141. 10.1080/07924259.1997.9672571

[16] Skinner LF, Macharet HKL, Coutinho R. Influence of upwelling and tropical environments on the breeding development of the intertidal barnacle *Tetraclita stalactifera* (Lamarck, 1818). Braz J Oceanogr. 2011;59: 349–356. 10.1590/S1679-87592011000400005

[17] Adamec D, O’Brien JJ. The seasonal upwelling in the Gulf of Guinea due to remote forcing. J Phys Oceanogr. 1978;8: 1050–1060. 10.1175/1520-0485(1978)008<1050:TSUITG>2.0.CO;2

[18] Lewis RK. Seasonal upwelling along the south-eastern coastline of South Australia. Mar Freshw Res. 1981;32: 843–854.

[19] Field JG, Shillington FA. Variability of the Benguela Current system The sea: the global coastal ocean: interdisciplinary regional studies and syntheses. Harvard University: Harvard University Press; 2006 pp. 835–863.

[20] García-Reyes M, Largier JL. Seasonality of coastal upwelling off central and northern California: new insights, including temporal and spatial variability. J Geophys Res Oceans. 2012;117: C03028 10.1029/2011JC007629

[21] Corbisier TN, Petti MAV, Soares LSH, Muto EY, Bromberg S, Valiela I. Trophic structure of benthic communities in the Cabo Frio upwelling system (southeastern Brazilian shelf): a temporal study using stable isotope analysis. Mar Ecol Prog Ser. 2014;512: 23–38. 10.3354/meps10947

[22] Soares LSH, Muto EY, Lopez JP, Clauzet GRV, Valiela I. Seasonal variability of δ^13^C and δ^15^N of fish and squid in the Cabo Frio upwelling system of the southwestern Atlantic. Mar Ecol Prog Ser. 2014;512: 9–21. 10.3354/meps10948

[23] Stenseth NC, Llope M, Anadón R, Ciannelli L, Chan K-S, Hjermann DØ, et al Seasonal plankton dynamics along a cross-shelf gradient. Proc R Soc Lond B Biol Sci. 2006;273: 2831–2838. 10.1098/rspb.2006.3658 PMC1664633 17015313

[24] Chenillat F, Rivière P, Capet X, Di Lorenzo E, Blanke B. North Pacific Gyre Oscillation modulates seasonal timing and ecosystem functioning in the California Current upwelling system. Geophys Res Lett. 2012;39: L01606 10.1029/2011GL049966

[25] Smith JR, Fong P, Ambrose RF. Spatial patterns in recruitment and growth of the mussel *Mytilus californianus* (Conrad) in southern and northern California, USA, two regions with differing oceanographic conditions. J Sea Res. 2009;61: 165–173. 10.1016/j.seares.2008.10.009

[26] De Léo FC, Pires-Vanin AMS. Benthic megafauna communities under the influence of the South Atlantic Central Water intrusion onto the Brazilian SE shelf: a comparison between an upwelling and a non-upwelling ecosystem. J Mar Syst. 2006;60: 268–284. 10.1016/j.jmarsys.2006.02.002

[27] Humborg C, Conley DJ, Rahm L, Wulff F, Cociasu A, Ittekkot V. Silicon retention in river basins: far-reaching effects on biogeochemistry and aquatic food webs in coastal marine environments. AMBIO J Hum Environ. 2000;29: 45–50. 10.1579/0044-7447-29.1.45

[28] Martin-Jézéquel V, Hildebrand M, Brzezinski MA. Silicon metabolism in diatoms: implications for growth. J Phycol. 2000;36: 821–840. 10.1046/j.1529-8817.2000.00019.x

[29] Tilstone G, Míguez B, Figueiras F, Fermín E. Diatom dynamics in a coastal ecosystem affected by upwelling: coupling between species succession, circulation and biogeochemical processes. Mar Ecol Prog Ser. 2000;205: 23–41. 10.3354/meps205023

[30] Cury P, Bakun A, Crawford RJM, Jarre A, Quiñones RA, Shannon LJ, et al Small pelagics in upwelling systems: patterns of interaction and structural changes in “wasp-waist” ecosystems. ICES J Mar Sci J Cons. 2000;57: 603–618. 10.1006/jmsc.2000.0712

[31] Chavez FP, Messié M. A comparison of eastern boundary upwelling ecosystems. Prog Oceanogr. 2009;83: 80–96. 10.1016/j.pocean.2009.07.032

[32] Andrews WRH, Hutchings L. Upwelling in the Southern Benguela Current. Prog Oceanogr. 1980;9: 1–81. 10.1016/0079-6611(80)90015-4

[33] Verheye Dua F, Lucas MI. Southern Benguela frontal region. 1. hydrology, phytoplankton and bacterioplankton. Mar Ecol Prog Ser. 1988;47: 271–280.

[34] Shannon LV, Hutchings L, Bailey GW, Shelton PA. Spatial and temporal distribution of chlorophyll in southern African waters as deduced from ship and satellite measurements and their implications for pelagic fisheries. South Afr J Mar Sci. 1984;2: 109–130. 10.2989/02577618409504363

[35] Pirini M, Manuzzi MP, Pagliarani A, Trombetti F, Borgatti AR, Ventrella V. Changes in fatty acid composition of *Mytilus galloprovincialis* (Lmk) fed on microalgal and wheat germ diets. Comp Biochem Physiol B Biochem Mol Biol. 2007;147: 616–626. 10.1016/j.cbpb.2007.04.003 17482494

[36] Allan EL, Ambrose ST, Richoux NB, Froneman PW. Determining spatial changes in the diet of nearshore suspension-feeders along the South African coastline: Stable isotope and fatty acid signatures. Estuar Coast Shelf Sci. 2010;87: 463–471. 10.1016/j.ecss.2010.02.004

[37] Xavier BM, Branch GM, Wieters EA. Abundance, growth and recruitment of *Mytilus galloprovincialis* on the west coast of South Africa in relation to upwelling. Mar Ecol Prog Ser. 2007;346: 189–201. 10.3354/meps07007

[38] Lutjeharms JRE, Cooper J, Roberts M. Upwelling at the inshore edge of the Agulhas Current. Cont Shelf Res. 2000;20: 737–761. 10.1016/S0278-4343(99)00092-8

[39] Gorokhova E, Hansson S. An experimental study on variations in stable carbon and nitrogen isotope fractionation during growth of *Mysis mixta* and *Neomysis integer*. Can J Fish Aquat Sci. 1999;56: 2203–2210. 10.1139/cjfas-56-11-2203

[40] Davenport J, Chen X. A comparison of methods for the assessment of condition in the mussel (*Mytilus edulis L*.). J Molluscan Stud. 1987;53: 293–297.

[41] Williams JR, Babcock RC. Assessment of size at maturity and gonad index methods for the scallop *Pecten novaezelandiae*. N Z J Mar Freshw Res. 2005;39: 851–864.

[42] Indarti E, Majid MIA, Hashim R, Chong A. Direct FAME synthesis for rapid total lipid analysis from fish oil and cod liver oil. J Food Compos Anal. 2005;18: 161–170. 10.1016/j.jfca.2003.12.007

[43] Ackman RG. The gas chromatograph in practical analyses of common and uncommon fatty acids for the 21st century. Anal Chim Acta. 2002;465: 175–192. 10.1016/S0003-2670(02)00098-3

[44] Budge SM, Iverson SJ, Koopman HN. Studying trophic ecology in marine ecosystems using fatty acids: a primer on analysis and interpretation. Mar Mammal Sci. 2006;22: 759–801. 10.1111/j.1748-7692.2006.00079.x

[45] Underwood AJ. Experiments in ecology: their logical design and interpretation using analysis of variance. Cambridge University Press; 1997.

[46] Anderson MJ. A new method for non-parametric multivariate analysis of variance. Austral Ecol. 2001;26: 32–46. 10.1111/j.1442-9993.2001.01070.pp.x

[47] Anderson M, Braak CT. Permutation tests for multi-factorial analysis of variance. J Stat Comput Simul. 2003;73: 85–113. 10.1080/00949650215733

[48] Clarke KR, Gorley RN. Primer V6: user manual—tutorial. Plymouth Marine Laboratory; 2006.

[49] Anderson M, Gorley R, Clarke K. PERMANOVA + for PRIMER: guide to software and statistical methods. PRIMER-E; 2008.

[50] Budge SM, Parrish CC. Lipid biogeochemistry of plankton, settling matter and sediments in Trinity Bay, Newfoundland. II. Fatty acids. Org Geochem. 1998; 1547–1559. 10.1016/S0146-6380(98)00177-6

[51] Dalsgaard J, St. John M, Kattner G, Müller-Navarra D, Hagen W. Fatty acid trophic markers in the pelagic marine environment Advances in Marine Biology. Academic Press; 2003 pp. 225–340. Available: http://www.sciencedirect.com/science/article/pii/S0065288103460057 10.1016/s0065-2881(03)46005-7 14601414

[52] Meeuwis JM, Lutjeharms JRE. Surface thermal characteristics of the Angola-Benguela front. South Afr J Mar Sci. 1990;9: 261–279. 10.2989/025776190784378772

[53] Demarcq H. Trends in primary production, sea surface temperature and wind in upwelling systems (1998–2007). Prog Oceanogr. 2009;83: 376–385. 10.1016/j.pocean.2009.07.022

[54] Pitcher GC, Brown PC, Mitchell-Innes BA. Spatio-temporal variability of phytoplankton in the southern Benguela upwelling system. South Afr J Mar Sci. 1992;12: 439–456. 10.2989/02577619209504717

[55] Shannon LV, Nelson G. The Benguela: large scale features and processes and system variability The South Atlantic. Springer Berlin Heidelberg; 1996 pp. 163–210. Available: http://link.springer.com/chapter/10.1007/978-3-642-80353-6_9

[56] Ezgeta-Balić D, Lojen S, Dolenec T, Žvab Rožič P, Dolenec M, Najdek M, et al Seasonal differences of stable isotope composition and lipid content in four bivalve species from the Adriatic Sea. Mar Biol Res. 2014;10: 625–634. 10.1080/17451000.2013.833338

[57] Basterretxea G, Arístegui J. Mesoscale variability in phytoplankton biomass distribution and photosynthetic parameters in the Canary-NW African coastal transition zone. Mar Ecol Prog Ser. 2000;197: 27–40. 10.3354/meps197027

[58] Suchanek TH, Geller JB, Kreiser BR, Mitton JB. Zoogeographic distributions of the sibling species *Mytilus galloprovincialis* and *M*. *trossulus (Bivalvia*: *Mytilidae*) and their hybrids in the North Pacific. Biol Bull. 1997;193: 187 10.2307/1542764 28575597

[59] Anestis A, Pörtner HO, Karagiannis D, Angelidis P, Staikou A, Michaelidis B. Response of *Mytilus galloprovincialis* (*L*.) to increasing seawater temperature and to marteliosis: metabolic and physiological parameters. Comp Biochem Physiol A Mol Integr Physiol. 2010;156: 57–66. 10.1016/j.cbpa.2009.12.018 20045485

[60] Tagliarolo M, McQuaid CD. Sub-lethal and sub-specific temperature effects are better predictors of mussel distribution than thermal tolerance. Mar Ecol Prog Ser. 2015;535: 145–159. 10.3354/meps11434

[61] Robinson T, Griffiths C, McQuaid C, Rius M. Marine alien species of South Africa—status and impacts. Afr J Mar Sci. 2005;27: 297–306. 10.2989/18142320509504088

[62] Fly EK, Hilbish TJ. Physiological energetics and biogeographic range limits of three congeneric mussel species. Oecologia. 2012;172: 35–46. 10.1007/s00442-012-2486-6 23064978

[63] Camacho AP, Labarta U, Beiras R. Growth of mussels (*Mytilus edulis galloprovincialis*) on cultivation rafts: influence of seed source, cultivation site and phytoplankton availability. Aquaculture. 1995;138: 349–362. 10.1016/0044-8486(95)01139-0

[64] Pampanin DM, Volpato E, Marangon I, Nasci C. Physiological measurements from native and transplanted mussel (*Mytilus galloprovincialis*) in the canals of Venice. Survival in air and condition index. Comp Biochem Physiol A Mol Integr Physiol. 2005;140: 41–52. 10.1016/j.cbpb.2004.10.016 15664311

[65] Field JG, Griffiths CL, Linley EA, Carter RA, Zoutendyk P. Upwelling in a nearshore marine ecosystem and its biological implications. Estuar Coast Mar Sci. 1980;11: 133–150. 10.1016/S0302-3524(80)80037-5

[66] Archambault P, McKindsey CW, Bourget E. Large-scale shoreline configuration influences phytoplankton concentration and mussel growth. Estuar Coast Shelf Sci. 1999;49: 193–208. 10.1006/ecss.1999.0481

[67] Giese AC, Hart MA, Smith AM, Cheung MA. Seasonal changes in body component indices and chemical composition in the pismo clam *Tivela stultorum*. Comp Biochem Physiol. 1967;22: 549–561. 10.1016/0010-406X(67)90617-2

[68] Maxwell MR, Hanlon RT. Female reproductive output in the squid *Loligo pealeii*: multiple egg clutches and implications for a spawning strategy. Mar Ecol Prog Ser. 2000;199: 159–170.

[69] van Erkom Schurink C, Griffiths C. A comparison of reproductive cycles and reproductive output in four southern African mussel species. Mar Ecol Prog Ser. 1991;76: 123–134. 10.3354/meps076123

[70] Porri F, McQuaid C, Radloff S. Spatio-temporal variability of larval abundance and settlement of *Perna perna*: differential delivery of mussels. Mar Ecol Prog Ser. 2006;315: 141–150. 10.3354/meps315141

[71] Alkanani T, Parrish CC, Thompson RJ, McKenzie CH. Role of fatty acids in cultured mussels, *Mytilus edulis*, grown in Notre Dame Bay, Newfoundland. J Exp Mar Biol Ecol. 2007;348: 33–45. 10.1016/j.jembe.2007.02.017

[72] Parrish CC, Abrajano TA, Budge SM, Helleur RJ, Hudson ED, Pulchan K, et al Lipid and phenolic biomarkers in marine ecosystems: analysis and applications In: Wangersky PJ, editor. Marine Chemistry. Springer Berlin Heidelberg; 2000 pp. 193–223. Available: http://link.springer.com/chapter/10.1007/10683826_8

[73] Sushchik NN. Role of essential fatty acids in trophometabolic interactions in the freshwater ecosystems (a review). Zhurnal Obshcheĭ Biol. 2008;69: 299–316. 18792646

[74] Arts MT, Ackman RG, Holub BJ. “Essential fatty acids” in aquatic ecosystems: a crucial link between diet and human health and evolution. Can J Fish Aquat Sci. 2001;58: 122–137. 10.1139/f00-224

[75] Kelly JR, Scheibling RE. Fatty acids as dietary tracers in benthic food webs. Mar Ecol Prog Ser. 2012;446: 1–22. 10.3354/meps09559

[76] Pollero RJ, Irazú CE, Brenner RR. Effect of sexual stages on lipids and fatty acids of *Diplodon delodontus*. Comp Biochem Physiol Part B Comp Biochem. 1983;76: 927–931. 10.1016/0305-0491(83)90414-5

[77] Soudant P, Moal J, Marty Y, Samain JF. Impact of the quality of dietary fatty acids on metabolism and the composition of polar lipid classes in female gonads of *Pecten maximus* (L.). J Exp Mar Biol Ecol. 1996;205: 149–163. 10.1016/S0022-0981(96)02608-1

[78] Kattner G, Hagen W. Lipids in marine copepods: latitudinal characteristics and perspective to global warming In: Kainz M, Brett MT, Arts MT, editors. Lipids in Aquatic Ecosystems. Springer New York; 2009 pp. 257–280. Available: http://link.springer.com/chapter/10.1007/978-0-387-89366-2_11

[79] Estefanell J, Socorro J, Izquierdo M, Roo J. Effect of two fresh diets and sexual maturation on the proximate and fatty acid profile of several tissues in *Octopus vulgaris*: specific retention of arachidonic acid in the gonads. Aquac Nutr. 2014;41 10.1111/anu.12163

[80] Blanchard G, Druart X, Kestemont P. Lipid content and fatty acid composition of target tissues in wild *Perca fluviatilis* females in relation to hepatic status and gonad maturation. J Fish Biol. 2005;66: 73–85. 10.1111/j.0022-1112.2005.00578.x

[81] Besnard J-Y, Lubet P, Nouvelot A. Seasonal variations of the fatty acid content of the neutral lipids and phospholipids in the female gonad of *Pecten maximus* L. Comp Biochem Physiol Part B Comp Biochem. 1989;93: 21–26. 10.1016/0305-0491(89)90210-1

[82] Napolitano GE, Ackman RG, Silva-Serra MA. Incorporation of dietary sterols by the sea scallop *Placopecten magellanicus* (Gmelin) fed on microalgae. Mar Biol. 1993;117: 647–654. 10.1007/BF00349777

[83] Brown PC, Field JG. Factors limiting phytoplankton production in a nearshore upwelling area. J Plankton Res. 1986;8: 55–68. 10.1093/plankt/8.1.55

[84] Wieters EA, Kaplan DM, Navarrete SA, Sotomayor A, Largier JL, Nielsen K, et al Alongshore and temporal variability in chlorophyll *a* concentration in Chilean nearshore waters. Mar Ecol Prog Ser. 2003;249: 93–105.

[85] Roughan M, Mace AJ, Largier JL, Morgan SG, Fisher JL, Carter ML. Subsurface recirculation and larval retention in the lee of a small headland: A variation on the upwelling shadow theme. J Geophys Res Oceans. 2005;110: 0–27. 10.1029/2005JC002898

[86] Nielsen KJ, Navarrete SA. Mesoscale regulation comes from the bottom-up: intertidal interactions between consumers and upwelling. Ecol Lett. 2004;7: 31–41. 10.1046/j.1461-0248.2003.00542.x

[87] Farrell TM, Bracher D, Roughgarden J. Cross-shelf transport causes recruitment to intertidal populations in central California. Limnol Oceanogr. 1991;36: 279–288. 10.4319/lo.1991.36.2.0279

[88] Wing SR, Largier J, Botsford L, Quinn J. Settlement and transport of benthic invertebrates in an intermittent upwelling region. Limnol Oceanogr. 1995;40: 316.

[89] Puccinelli E, Noyon M, McQuaid C. Hierarchical effects of biogeography and upwelling shape the dietary signatures of benthic filter feeders. Mar Ecol Prog Ser. 2016; 10.3354/meps11567

[90] Shannon LV, Mostert SA, Walters NM, Anderson FP. Chlorophyll concentrations in the southern Benguela Current region as determined by satellite (Nimbus-7 coastal zone colour scanner). J Plankton Res. 1983;5: 565–583. 10.1093/plankt/5.4.565

