# Supplementary figures and images for: Spatio-Temporal Variation in Effects of Upwelling on the Fatty Acid Composition of Benthic Filter Feeders in the Southern Benguela Ecosystem: Not All Upwelling Is Equal
Source: PLoS One. 2016 Aug 29;11(8):e0161919. doi: 10.1371/journal.pone.0161919 (PMC5003371; doi:10.1371/journal.pone.0161919)

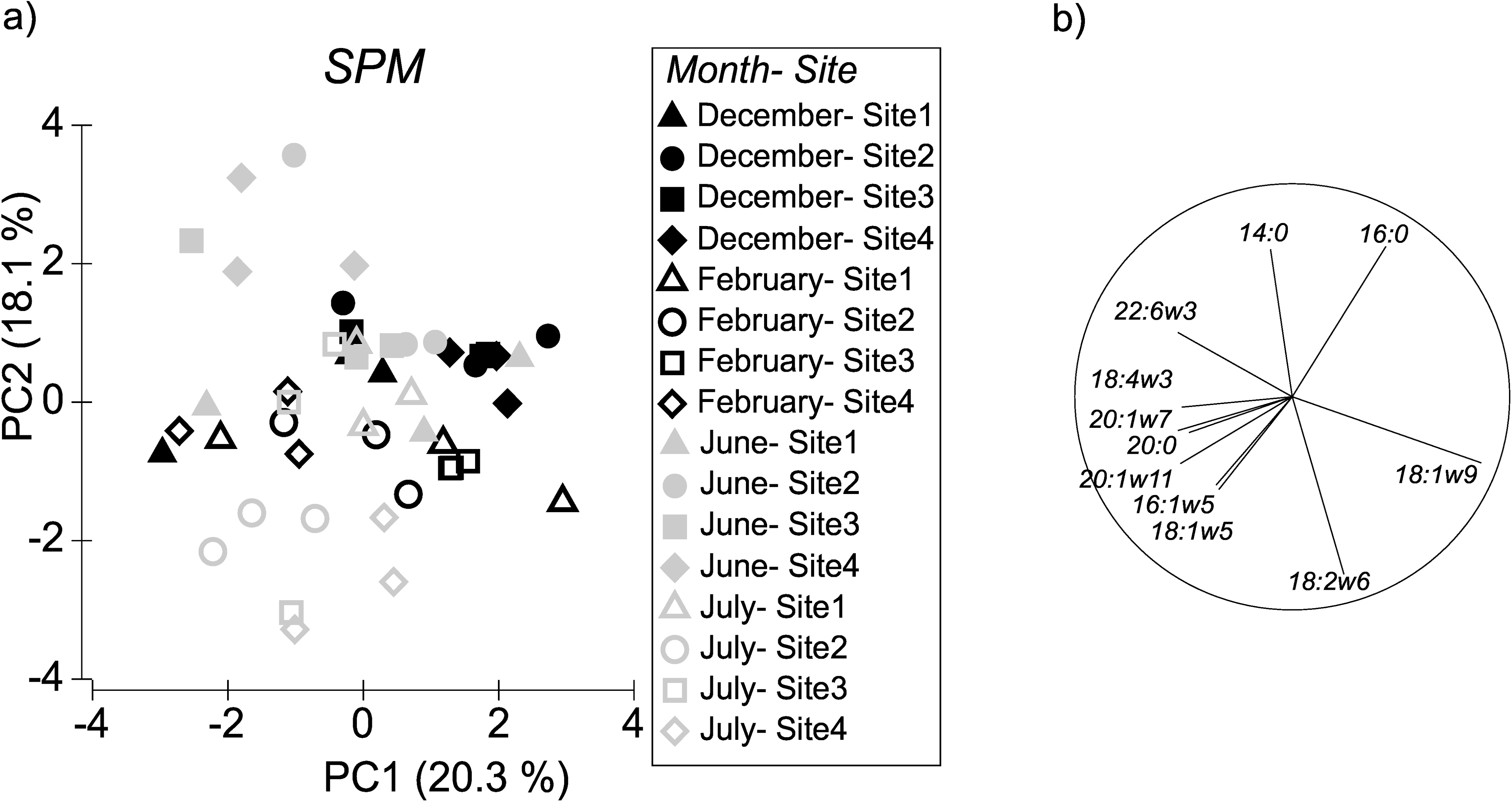

Supplement: S1 Fig — a) PCA conducted on the fatty acid composition of suspended organic matter (SPM) collected from intertidal rocky shores at four sites during the four sampling events. Each symbol represents a single replicate. Upwelling did not have an effect on the fatty acid signatures of samples from February and thus we show a single PCA with the samples of all months together. b) Eigenvalues for each of the factors (fatty acids). The circle corresponds to eigenvalues of −1 to 1. For clarity, only fatty acids with eigenvalues > 0.5 are shown. (TIF) [file pone.0161919.s001.tif]

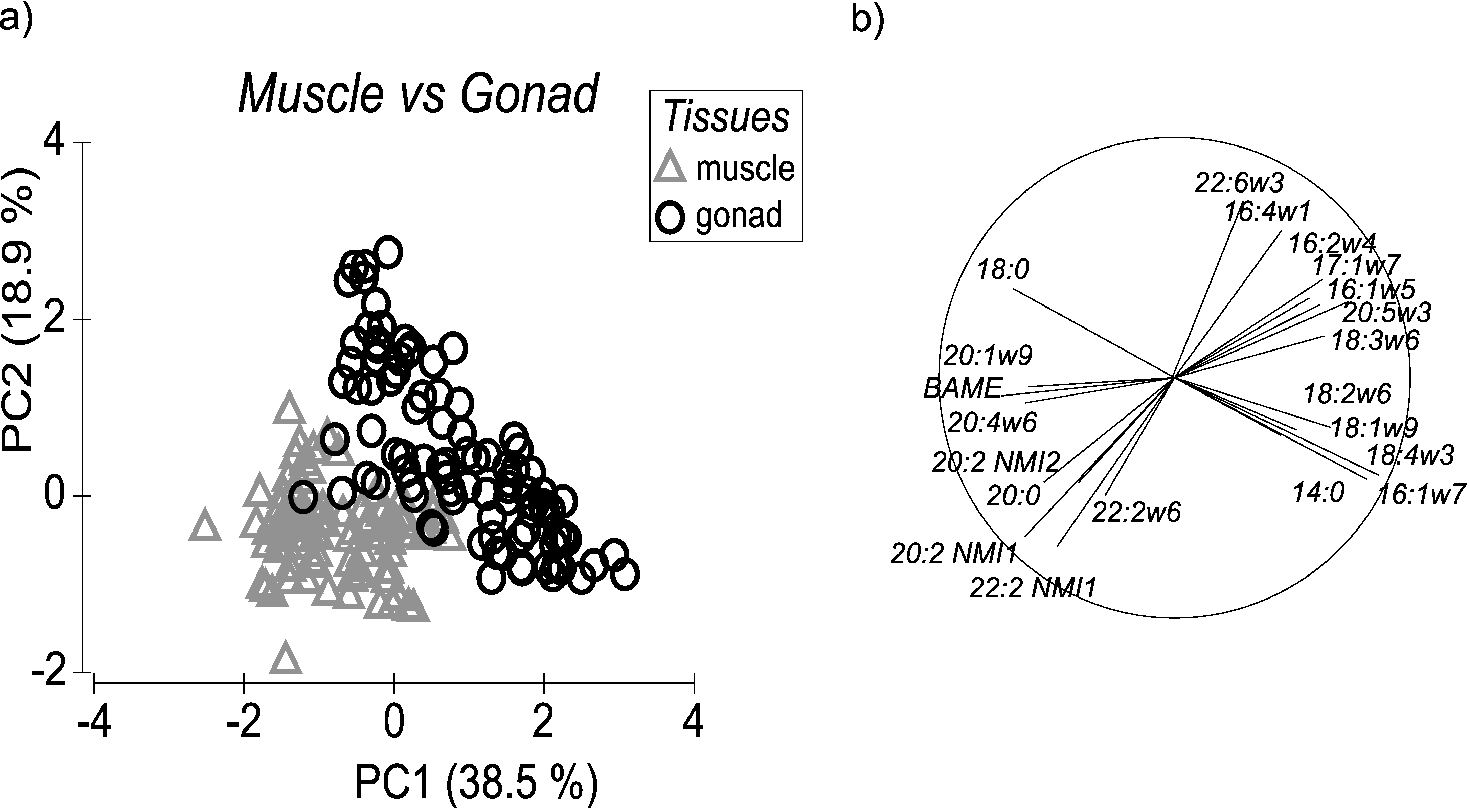

Supplement: S2 Fig — a) PCA conducted on the adductor muscle and gonad tissues of Mytilus galloprovincialis collected at intertidal rocky shores at each site during the four sampling events. Each symbol represents a single replicate. b) Eigenvalues of each of the factors (fatty acids). The circle corresponds to eigenvalues of −1 to 1. For clarity, only fatty acids with eigenvalues > 0.5 are shown. (TIF) [file pone.0161919.s002.tif]

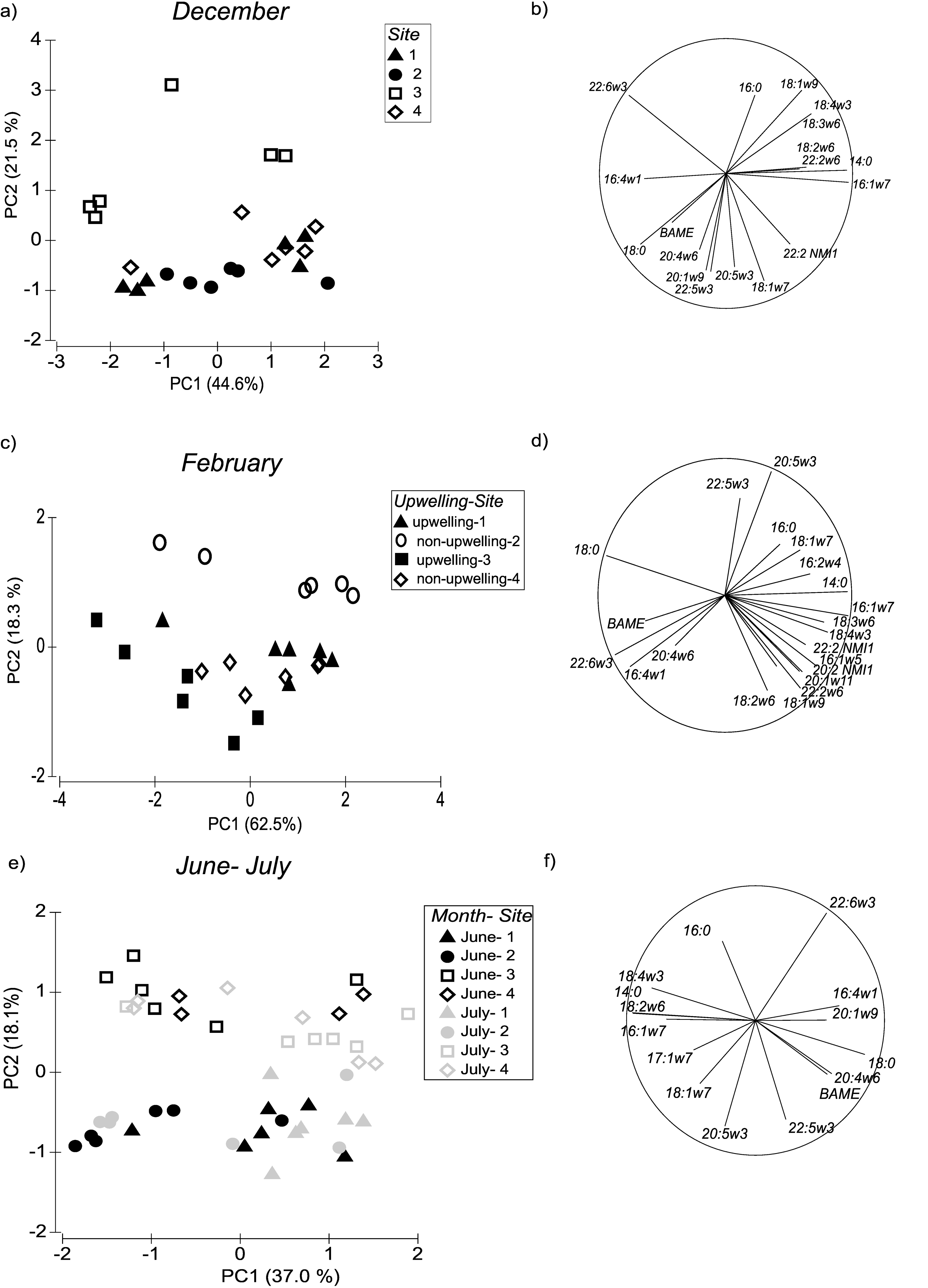

Supplement: S3 Fig — PCA conducted on the fatty acid composition of gonad tissue of Mytilus galloprovincialis collected from intertidal rocky shores at four sites during a) December c) February e) June and July. Each symbol represents a single replicate. b), d) and f) Eigenvalues of each of the factors (fatty acids). The circle corresponds to eigenvalues of −1 to 1. For clarity, only fatty acids with eigenvalues > 0.5 are shown. (TIF) [file pone.0161919.s003.tif]
