# Supplementary material for: Spatio-Temporal Variation in Effects of Upwelling on the Fatty Acid Composition of Benthic Filter Feeders in the Southern Benguela Ecosystem: Not All Upwelling Is Equal
Source: PLoS One. 2016 Aug 29;11(8):e0161919. doi: 10.1371/journal.pone.0161919 (PMC5003371; doi:10.1371/journal.pone.0161919)
Supplement: S1 File — (DOCX) [file pone.0161919.s004.docx]

**Supplementary material**

**Table A** Categorization of sites as experiencing upwelling or non-upwelling conditions on each sampling occasion, based on in situ temperature logger data. NA = non-available

| Site | December | February | June | July |
| --- | --- | --- | --- | --- |
| 1 | NA | upwelling | non- upwelling | non- upwelling |
| 2 | NA | non- upwelling | non- upwelling | non- upwelling |
| 3 | NA | upwelling | non- upwelling | non- upwelling |
| 4 | NA | non- upwelling | non- upwelling | non- upwelling |

**Table B** Fatty acid composition of adductor muscles of Mytilus galloprovincialis collected during 4 sampling events and across four sites along the South African west coast. The values are percentages expressed as mean ± standard deviation (n = 6 per site). Only FA contributing > 1 % to total fatty acids are displayed. PUFA = Polyunsaturated Fatty Acids, MUFA= Monounsaturated Fatty Acids, SFA= Saturated Fatty Acids, EFA = Essential Fatty Acids (20:4w6, 20:5w3 and 22:6w3), NMI = Non-methylene-interrupted Fatty Acid, BAME = Bacterial Fatty Acids.

**Table** **C** Fatty acid composition of gonads of Mytilus galloprovincialis collected in December, February, June and July at four sampling sites along the South African west coast. The values are percentages expressed as mean ± standard deviation (n = 6 per site). Only FA contributing > 1 % to total fatty acids are displayed. PUFA = Polyunsaturated Fatty Acids, MUFA= Monounsaturated Fatty Acids, SFA= Saturated Fatty Acids, EFA = Essential Fatty Acids (20:4w6, 20:5w3 and 22:6w3), NMI = Non-methylene-interrupted Fatty Acid, BAME = Bacterial Fatty Acids.

**Table D** Table reporting the locations sampled with their geographic coordinates, and the sampling dates.

| **Site** | **Latitude** | **Longitude** | **t0- December** | **t1- February** | **t2- June** | **t3- July** |
| --- | --- | --- | --- | --- | --- | --- |
| 1- Llandudno | 34° 0'16.67"S | 18°20'25.26"E | 10/12/2012 | 08/02/2013 | 12/06/2013 | 08/07/2013 |
| 2- Bloubergstrand | 33°48'22.30"S | 18°27'51.42"E | 10/12/2012 | 08/02/2013 | 12/06/2013 | 08/07/2013 |
| 3- Paternoster | 32°48'30.70"S | 17°52'57.78"E | 11/12/2012 | 09/02/2013 | 13/06/2013 | 09/07/2013 |
| 4- Elandsbaai | 32°19'2.44"S | 18°19'25.03"E | 11/12/2012 | 09/02/2013 | 13/06/2013 | 09/07/2013 |

**Table E** Mean (± standard deviation) of Condition Index and Gonad Index (n = 26 and n = 6 for CI and GI, respectively) of *Mytilus galloprovincialis* at the four sites and across the four months sampled. Sites 1 and 3 were upwelling sites while sites 2 and 4 non-upwelling sites in February only. In June and July all sited were categorized as non-upwelling sites. In December we were not able to classify the sites.

|  | **Month** | **Site 1** | | | **Site 2** | | | **Site 3** | | | **Site 4** | | |
| --- | --- | --- | --- | --- | --- | --- | --- | --- | --- | --- | --- | --- | --- |
|  |  | mean |  | SD | mean |  | SD | mean |  | SD | mean |  | SD |
| **Condition Index** | December | 3.87 | ± | 0.76 | 4.83 | ± | 1.15 | 12.82 | ± | 3.19 | 6.03 | ± | 1.83 |
|  | February | 4.48 | ± | 1.22 | 6.3 | ± | 2.65 | 13.5 | ± | 5.61 | 11.42 | ± | 2.16 |
|  | June | 4.22 | ± | 1.75 | 9.01 | ± | 3.12 | 13.52 | ± | 3.59 | 9.09 | ± | 2.8 |
|  | July | 3.64 | ± | 1.16 | 9.08 | ± | 2.26 | 11.44 | ± | 2.69 | 7.53 | ± | 2.52 |
| **Gonad Index** | December | 15.66 | ± | 3.47 | 18.65 | ± | 3.6 | 32.73 | ± | 6.42 | 18.34 | ± | 4.86 |
|  | February | 19.86 | ± | 6.94 | 21.6 | ± | 3.91 | 30.23 | ± | 10.98 | 28.25 | ± | 6.74 |
|  | June | 16.44 | ± | 5.05 | 25.22 | ± | 5.31 | 31.92 | ± | 10.21 | 28.88 | ± | 6.45 |
|  | July | 7.78 | ± | 4.48 | 16.17 | ± | 9.38 | 38.48 | ± | 7.60 | 35.96 | ± | 15.17 |

**Table F** Raw data used to calculate the Condition Index and Gonad Index of *Mytilus galloprovincialis* at the four sites and across the four months sampled.

| **Month** | **Site** | **Shell Length (cm)** | **Weight Shell (mg)** | **Weight Gonads (mg)** | **Total Body Weight (mg)** | **CI** | **GI** |
| --- | --- | --- | --- | --- | --- | --- | --- |
| December | 1 | 6.00 | 9740.10 | 92.30 | 430.10 | 4.42 | 21.46 |
| December | 1 | 6.50 | 12658.30 | 66.90 | 504.10 | 3.98 | 13.27 |
| December | 1 | 5.50 | 8619.10 | 66.40 | 479.70 | 5.57 | 13.84 |
| December | 1 | 5.10 | 8561.90 | 129.70 | 641.90 | 7.50 | 20.21 |
| December | 1 | 5.10 | 5513.70 | 53.00 | 326.20 | 5.92 | 16.25 |
| December | 1 | 5.60 | 4739.70 | 60.50 | 337.70 | 7.12 | 17.92 |
| December | 1 | 5.30 | 6879.70 | 49.50 | 374.80 | 5.45 | 13.21 |
| December | 1 | 5.50 | 6292.30 | 41.70 | 408.90 | 6.50 | 10.20 |
| December | 1 | 5.20 | 5624.80 | 56.80 | 439.50 | 7.81 | 12.92 |
| December | 1 | 5.40 | 4297.50 | 44.20 | 255.20 | 5.94 | 17.32 |
| December | 2 | 6.40 | 12472.80 | 372.40 | 1002.10 | 8.03 | 37.16 |
| December | 2 | 6.10 | 10993.40 | 106.00 | 805.20 | 7.32 | 13.16 |
| December | 2 | 6.20 | 11737.30 | 233.60 | 1102.10 | 9.39 | 21.20 |
| December | 2 | 6.70 | 11680.40 | 148.50 | 943.20 | 8.08 | 15.74 |
| December | 2 | 6.20 | 11212.00 | 104.80 | 787.90 | 7.03 | 13.30 |
| December | 2 | 5.70 | 7378.40 | 188.70 | 704.30 | 9.55 | 26.79 |
| December | 2 | 5.70 | 9115.70 | 136.80 | 614.90 | 6.75 | 22.25 |
| December | 2 | 5.60 | 10179.40 | 129.60 | 607.20 | 5.96 | 21.34 |
| December | 2 | 5.60 | 8316.10 | 72.60 | 392.80 | 4.72 | 18.48 |
| December | 2 | 5.60 | 5684.90 | 72.40 | 393.40 | 6.92 | 18.40 |
| December | 3 | 6.60 | 11231.10 | 170.30 | 1151.10 | 10.25 | 14.79 |
| December | 3 | 5.90 | 10169.70 | 508.90 | 1145.45 | 11.26 | 44.43 |
| December | 3 | 5.90 | 11060.10 | 398.50 | 1237.20 | 11.19 | 32.21 |
| December | 3 | 5.80 | 8673.90 | 543.60 | 1524.20 | 17.57 | 35.66 |
| December | 3 | 6.20 | 8944.70 | 528.90 | 1475.10 | 16.49 | 35.86 |
| December | 3 | 5.40 | 6241.80 | 286.90 | 827.10 | 13.25 | 34.69 |
| December | 3 | 6.00 | 7544.40 | 441.00 | 1130.40 | 14.98 | 39.01 |
| December | 3 | 5.70 | 6888.50 | 379.80 | 1129.20 | 16.39 | 33.63 |
| December | 3 | 4.90 | 5289.80 | 330.30 | 996.90 | 18.85 | 33.13 |
| December | 3 | 5.60 | 4808.60 | 233.80 | 605.10 | 12.58 | 38.64 |
| December | 4 | 6.10 | 10399.60 | 143.80 | 930.40 | 8.95 | 15.46 |
| December | 4 | 6.00 | 9654.30 | 76.80 | 632.51 | 6.55 | 12.14 |
| December | 4 | 6.60 | 11692.70 | 186.60 | 1052.40 | 9.00 | 17.73 |
| December | 4 | 5.70 | 9993.60 | 143.90 | 1100.70 | 11.01 | 13.07 |
| December | 4 | 5.70 | 8833.10 | 83.40 | 506.90 | 5.74 | 16.45 |
| December | 4 | 5.40 | 9979.90 | 158.80 | 768.50 | 7.70 | 20.66 |
| December | 4 | 5.00 | 7169.60 | 201.80 | 837.70 | 11.68 | 24.09 |
| December | 4 | 5.10 | 6337.60 | 67.60 | 477.90 | 7.54 | 14.15 |
| December | 4 | 5.10 | 7049.40 | 155.40 | 854.65 | 12.12 | 18.18 |
| December | 4 | 5.10 | 5902.20 | 93.50 | 645.80 | 10.94 | 14.48 |
| February | 1 | 6.30 | 9167.00 | 172.20 | 735.70 | 8.03 | 23.41 |
| February | 1 | 5.80 | 10050.40 | 49.10 | 487.30 | 4.85 | 10.08 |
| February | 1 | 4.90 | 7185.40 | 81.10 | 501.10 | 6.97 | 16.18 |
| February | 1 | 5.90 | 10980.80 | 119.40 | 768.60 | 7.00 | 15.53 |
| February | 1 | 6.30 | 12416.60 | 404.50 | 1160.40 | 9.35 | 34.86 |
| February | 1 | 5.20 | 6692.60 | 60.60 | 348.10 | 5.20 | 17.41 |
| February | 1 | 5.40 | 8384.60 | 70.30 | 450.60 | 5.37 | 15.60 |
| February | 1 | 6.00 | 8331.80 | 142.20 | 557.80 | 6.69 | 25.49 |
| February | 1 | 5.30 | 4575.10 | 35.18 | 246.90 | 5.40 | 14.25 |
| February | 1 | 5.60 | 6260.90 | 90.30 | 443.90 | 7.09 | 20.34 |
| February | 2 | 5.80 | 8226.30 | 307.70 | 1139.10 | 13.85 | 27.01 |
| February | 2 | 5.40 | 6420.10 | 239.40 | 897.80 | 13.98 | 26.67 |
| February | 2 | 6.40 | 10456.90 | 94.05 | 660.00 | 6.31 | 20.45 |
| February | 2 | 5.10 | 5363.60 | 101.90 | 559.10 | 10.42 | 18.23 |
| February | 2 | 5.10 | 5832.20 | 218.90 | 779.60 | 13.37 | 28.08 |
| February | 2 | 5.20 | 7338.40 | 87.60 | 545.50 | 7.43 | 16.06 |
| February | 2 | 5.20 | 9049.60 | 147.00 | 709.90 | 7.84 | 20.71 |
| February | 2 | 5.00 | 5944.00 | 70.00 | 473.70 | 7.97 | 14.78 |
| February | 2 | 5.50 | 9102.30 | 50.60 | 454.30 | 4.99 | 11.14 |
| February | 2 | 5.00 | 5452.10 | 103.80 | 487.50 | 8.94 | 21.29 |
| February | 3 | 5.00 | 5940.40 | 81.20 | 506.70 | 8.53 | 16.03 |
| February | 3 | 5.80 | 10537.30 | 150.25 | 483.70 | 4.59 | 31.06 |
| February | 3 | 5.40 | 6743.70 | 479.60 | 945.70 | 14.02 | 50.71 |
| February | 3 | 5.10 | 10468.70 | 199.00 | 915.00 | 8.74 | 21.75 |
| February | 3 | 5.00 | 5140.70 | 219.50 | 643.10 | 12.51 | 34.13 |
| February | 3 | 4.80 | 4653.10 | 160.80 | 605.70 | 13.02 | 26.55 |
| February | 3 | 5.30 | 5208.30 | 178.80 | 774.00 | 14.86 | 23.10 |
| February | 3 | 5.30 | 6889.20 | 435.00 | 1133.90 | 16.46 | 38.36 |
| February | 3 | 5.40 | 6159.80 | 121.32 | 424.20 | 6.89 | 28.60 |
| February | 3 | 4.90 | 3651.50 | 105.10 | 336.50 | 9.22 | 31.23 |
| February | 4 | 6.20 | 10078.90 | 114.90 | 581.90 | 5.77 | 19.75 |
| February | 4 | 5.70 | 9354.50 | 83.80 | 550.40 | 5.88 | 15.23 |
| February | 4 | 5.80 | 8633.30 | 167.40 | 654.20 | 7.58 | 25.59 |
| February | 4 | 6.20 | 12070.90 | 130.70 | 616.60 | 5.11 | 21.20 |
| February | 4 | 6.30 | 11066.10 | 134.50 | 716.30 | 6.47 | 18.78 |
| February | 4 | 4.80 | 7251.30 | 255.60 | 688.80 | 9.50 | 37.11 |
| February | 4 | 5.00 | 6273.10 | 199.30 | 749.30 | 11.94 | 26.60 |
| February | 4 | 5.10 | 7405.50 | 186.52 | 495.00 | 6.68 | 37.68 |
| February | 4 | 4.70 | 6235.00 | 128.40 | 616.20 | 9.88 | 20.84 |
| February | 4 | 4.60 | 5468.50 | 162.70 | 571.90 | 10.46 | 28.45 |
| June | 1 | 5.10 | 7235.00 | 71.60 | 446.90 | 6.18 | 16.02 |
| June | 1 | 4.70 | 6352.00 | 144.70 | 697.30 | 10.98 | 20.75 |
| June | 1 | 4.60 | 6854.00 | 35.00 | 324.00 | 4.73 | 10.80 |
| June | 1 | 5.20 | 5425.00 | 17.80 | 266.40 | 4.91 | 6.68 |
| June | 1 | 4.80 | 5143.10 | 33.40 | 308.10 | 5.99 | 10.84 |
| June | 1 | 4.90 | 4835.00 | 57.40 | 309.90 | 6.41 | 18.52 |
| June | 1 | 4.70 | 4657.00 | 127.20 | 612.00 | 13.14 | 20.78 |
| June | 1 | 5.40 | 5735.00 | 32.20 | 253.30 | 4.42 | 12.71 |
| June | 1 | 4.50 | 5935.00 | 86.20 | 388.50 | 6.55 | 22.19 |
| June | 1 | 4.40 | 6345.00 | 81.70 | 325.20 | 5.13 | 25.12 |
| June | 2 | 5.30 | 5519.10 | 193.90 | 1412.00 | 13.73 | 25.58 |
| June | 2 | 5.00 | 6336.30 | 189.80 | 2217.56 | 35.00 | 8.56 |
| June | 2 | 5.30 | 9301.00 | 294.70 | 3096.31 | 33.29 | 9.52 |
| June | 2 | 5.50 | 6924.80 | 30.50 | 389.53 | 5.63 | 7.83 |
| June | 2 | 6.10 | 8890.60 | 392.10 | 1225.21 | 13.78 | 32.00 |
| June | 2 | 5.30 | 9859.20 | 112.40 | 449.10 | 4.56 | 25.03 |
| June | 2 | 5.10 | 7635.10 | 267.90 | 931.40 | 12.20 | 28.76 |
| June | 2 | 5.30 | 6426.40 | 67.30 | 377.70 | 5.88 | 17.82 |
| June | 2 | 5.30 | 6373.60 | 144.10 | 664.40 | 10.42 | 21.69 |
| June | 2 | 5.00 | 6105.70 | 185.80 | 599.30 | 9.82 | 31.00 |
| June | 3 | 6.40 | 11343.10 | 747.00 | 1412.90 | 12.46 | 52.87 |
| June | 3 | 5.70 | 8194.20 | 120.90 | 632.70 | 7.72 | 19.11 |
| June | 3 | 5.10 | 7516.20 | 268.70 | 792.00 | 10.54 | 33.93 |
| June | 3 | 4.90 | 5773.80 | 449.00 | 1059.10 | 18.34 | 42.39 |
| June | 3 | 5.20 | 7638.60 | 462.00 | 1245.23 | 16.30 | 37.10 |
| June | 3 | 5.00 | 6603.60 | 257.80 | 842.60 | 12.76 | 30.60 |
| June | 3 | 5.20 | 5479.30 | 97.70 | 499.80 | 9.12 | 19.55 |
| June | 3 | 5.50 | 6283.70 | 229.30 | 1108.10 | 17.63 | 20.69 |
| June | 3 | 5.70 | 6686.80 | 225.80 | 691.70 | 10.34 | 32.64 |
| June | 3 | 5.20 | 5044.80 | 245.70 | 809.10 | 16.04 | 30.37 |
| June | 4 | 5.90 | 9256.00 | 102.00 | 341.80 | 3.69 | 29.84 |
| June | 4 | 5.00 | 7181.60 | 171.80 | 821.55 | 11.44 | 20.91 |
| June | 4 | 5.80 | 12651.60 | 491.40 | 1521.23 | 12.02 | 32.30 |
| June | 4 | 5.90 | 8349.00 | 271.50 | 929.60 | 11.13 | 29.21 |
| June | 4 | 4.80 | 7209.60 | 223.10 | 760.50 | 10.55 | 29.34 |
| June | 4 | 5.10 | 4120.20 | 192.80 | 745.23 | 18.09 | 25.87 |
| June | 4 | 5.20 | 6132.90 | 239.70 | 885.56 | 14.44 | 27.07 |
| June | 4 | 5.10 | 6736.60 | 324.30 | 901.60 | 13.38 | 35.97 |
| June | 4 | 5.00 | 7010.20 | 132.50 | 567.20 | 8.09 | 23.36 |
| June | 4 | 4.80 | 5366.70 | 179.70 | 658.00 | 12.26 | 27.31 |
| July | 1 | 5.20 | 7691.10 | 21.90 | 308.20 | 4.01 | 7.11 |
| July | 1 | 5.50 | 7204.30 | 60.40 | 542.12 | 7.52 | 11.14 |
| July | 1 | 5.20 | 8493.20 | 23.50 | 285.20 | 3.36 | 8.24 |
| July | 1 | 5.40 | 6741.90 | 58.40 | 379.60 | 5.63 | 15.38 |
| July | 1 | 5.20 | 9545.60 | 26.40 | 546.23 | 5.72 | 4.83 |
| July | 1 | 4.90 | 4827.35 | 30.20 | 255.70 | 5.30 | 11.81 |
| July | 1 | 4.60 | 4331.90 | 8.60 | 187.80 | 4.34 | 4.58 |
| July | 1 | 4.50 | 4422.60 | 9.50 | 131.50 | 2.97 | 7.22 |
| July | 1 | 4.30 | 1605.40 | 5.00 | 168.20 | 10.48 | 2.97 |
| July | 1 | 4.10 | 3568.70 | 25.12 | 210.90 | 5.91 | 11.91 |
| July | 2 | 5.20 | 8006.90 | 193.70 | 844.10 | 10.54 | 22.95 |
| July | 2 | 5.30 | 9522.10 | 290.70 | 1083.40 | 11.38 | 26.83 |
| July | 2 | 5.90 | 10333.20 | 154.12 | 772.30 | 7.47 | 19.96 |
| July | 2 | 5.60 | 11526.30 | 19.70 | 581.80 | 5.05 | 3.39 |
| July | 2 | 5.90 | 9622.90 | 187.40 | 1038.80 | 10.80 | 18.04 |
| July | 2 | 4.60 | 5463.30 | 94.30 | 380.60 | 6.97 | 24.78 |
| July | 2 | 4.30 | 5225.20 | 91.10 | 388.70 | 7.44 | 23.44 |
| July | 2 | 4.30 | 4195.60 | 33.70 | 202.40 | 4.82 | 16.65 |
| July | 2 | 4.20 | 4465.20 | 16.60 | 219.60 | 4.92 | 7.56 |
| July | 2 | 4.10 | 3722.70 | 55.90 | 309.40 | 8.31 | 18.07 |
| July | 3 | 5.00 | 6578.10 | 356.40 | 940.50 | 14.30 | 37.89 |
| July | 3 | 5.10 | 9501.00 | 359.60 | 854.25 | 8.99 | 42.10 |
| July | 3 | 5.40 | 7214.90 | 211.30 | 251.80 | 3.49 | 83.92 |
| July | 3 | 5.60 | 6958.20 | 205.90 | 1044.90 | 15.02 | 19.71 |
| July | 3 | 4.80 | 6202.60 | 173.90 | 625.32 | 10.08 | 27.81 |
| July | 3 | 5.30 | 5766.40 | 189.10 | 723.90 | 12.55 | 26.12 |
| July | 3 | 5.00 | 5623.50 | 189.50 | 581.80 | 10.35 | 32.57 |
| July | 3 | 5.00 | 5665.40 | 154.30 | 575.60 | 10.16 | 26.81 |
| July | 1 | 4.80 | 4985.25 | 200.10 | 645.90 | 12.96 | 30.98 |
| July | 3 | 4.60 | 4713.70 | 195.90 | 604.40 | 12.82 | 32.41 |
| July | 4 | 3.80 | 4083.60 | 96.40 | 340.50 | 8.34 | 28.31 |
| July | 4 | 3.80 | 2461.50 | 17.60 | 153.50 | 6.24 | 11.47 |
| July | 4 | 4.30 | 3187.40 | 35.24 | 200.90 | 6.30 | 17.54 |
| July | 4 | 4.20 | 2946.20 | 34.40 | 236.10 | 8.01 | 14.57 |
| July | 4 | 4.20 | 3351.70 | 71.00 | 350.90 | 10.47 | 20.23 |
| July | 4 | 7.10 | 16535.70 | 435.90 | 1415.70 | 8.56 | 30.79 |
| July | 4 | 5.50 | 8302.50 | 95.60 | 535.80 | 6.45 | 17.84 |
| July | 4 | 5.40 | 7680.20 | 165.80 | 425.12 | 5.54 | 39.00 |
| July | 4 | 6.00 | 10827.40 | 20.30 | 212.47 | 1.96 | 9.55 |
| July | 4 | 5.80 | 9715.80 | 338.70 | 371.80 | 5.89 | 59.23 |

**Table G** Daily mean (± standard deviation) of the temperature values recorded by data loggers (n = 4 per site) that were deployed during low tide at each site for the duration of the investigation.

| **Date** | **Site 1** | | | **Site 2** | | | **Site 3** | | | **Site 4** | | |
| --- | --- | --- | --- | --- | --- | --- | --- | --- | --- | --- | --- | --- |
| 12/12/2012 | 10.43 | ± | 0.15 | 16.38 | ± | 0.64 | 12.31 | ± | 0.18 | 17.17 | ± | 0.18 |
| 13/12/2012 | 12.33 | ± | 0.41 | 15.50 | ± | 0.80 | 13.66 | ± | 0.14 | 15.33 | ± | 0.14 |
| 14/12/2012 | 13.50 | ± | 0.17 | 17.47 | ± | 0.48 | 15.86 | ± | 0.11 | 18.00 | ± | 0.11 |
| 15/12/2012 | 14.70 | ± | 0.25 | 18.75 | ± | 0.48 | 16.41 | ± | 0.29 | 19.17 | ± | 0.29 |
| 16/12/2012 | 14.50 | ± | 0.20 | 17.71 | ± | 0.61 | 17.62 | ± | 0.21 | 20.17 | ± | 0.21 |
| 17/12/2012 | 12.67 | ± | 0.78 | 18.86 | ± | 0.75 | 14.10 | ± | 0.96 | 21.95 | ± | 0.96 |
| 18/12/2012 | 12.10 | ± | 0.99 | 18.25 | ± | 0.79 | 13.81 | ± | 0.85 | 21.45 | ± | 0.85 |
| 19/12/2012 | 13.97 | ± | 0.68 | 17.57 | ± | 0.68 | 11.81 | ± | 0.64 | 20.88 | ± | 0.64 |
| 20/12/2012 | 11.50 | ± | 0.67 | 17.72 | ± | 0.43 | 12.36 | ± | 0.11 | 20.43 | ± | 0.11 |
| 21/12/2012 | 14.46 | ± | 0.98 | 18.57 | ± | 0.55 | 14.71 | ± | 0.14 | 16.93 | ± | 0.14 |
| 22/12/2012 | 16.53 | ± | 0.30 | 21.11 | ± | 0.10 | 16.77 | ± | 0.57 | 19.68 | ± | 0.57 |
| 23/12/2012 | 15.63 | ± | 0.48 | 19.50 | ± | 0.23 | 15.96 | ± | 0.68 | 20.28 | ± | 0.68 |
| 24/12/2012 | 14.90 | ± | 0.19 | 19.85 | ± | 0.35 | 16.07 | ± | 0.25 | 18.95 | ± | 0.25 |
| 25/12/2012 | 16.53 | ± | 0.32 | 20.78 | ± | 0.10 | 19.17 | ± | 0.14 | 20.15 | ± | 0.14 |
| 26/12/2012 | 15.93 | ± | 0.49 | 21.43 | ± | 0.15 | 18.37 | ± | 0.33 | 21.30 | ± | 0.33 |
| 27/12/2012 | 16.90 | ± | 0.15 | 21.07 | ± | 0.16 | 17.97 | ± | 0.14 | 20.30 | ± | 0.14 |
| 28/12/2012 | 17.43 | ± | 0.09 | 21.51 | ± | 0.17 | 17.92 | ± | 0.22 | 21.08 | ± | 0.22 |
| 29/12/2012 | 18.07 | ± | 0.15 | 21.33 | ± | 0.30 | 19.32 | ± | 0.00 | 21.70 | ± | 0.00 |
| 30/12/2012 | 18.10 | ± | 0.19 | 20.93 | ± | 0.42 | 18.42 | ± | 0.45 | 21.63 | ± | 0.45 |
| 31/12/2012 | 10.83 | ± | 0.51 | 19.65 | ± | 0.38 | 11.76 | ± | 0.27 | 17.00 | ± | 0.27 |
| 01/01/2013 | 10.53 | ± | 0.30 | 17.75 | ± | 0.38 | 11.41 | ± | 0.29 | 15.30 | ± | 0.29 |
| 02/01/2013 | 11.07 | ± | 0.35 | 16.23 | ± | 0.60 | 12.26 | ± | 0.33 | 15.93 | ± | 0.33 |
| 03/01/2013 | 10.83 | ± | 0.17 | 16.58 | ± | 0.53 | 14.86 | ± | 0.21 | 16.48 | ± | 0.21 |
| 04/01/2013 | 11.37 | ± | 0.53 | 16.81 | ± | 0.50 | 13.76 | ± | 0.82 | 16.63 | ± | 0.82 |
| 05/01/2013 | 11.07 | ± | 0.60 | 16.69 | ± | 0.32 | 13.51 | ± | 0.41 | 15.85 | ± | 0.41 |
| 06/01/2013 | 11.07 | ± | 0.28 | 17.11 | ± | 0.57 | 11.06 | ± | 0.35 | 15.10 | ± | 0.35 |
| 07/01/2013 | 10.97 | ± | 0.40 | 16.90 | ± | 0.23 | 11.36 | ± | 0.33 | 14.73 | ± | 0.33 |
| 08/01/2013 | 10.97 | ± | 0.30 | 17.95 | ± | 0.38 | 11.36 | ± | 0.45 | 14.68 | ± | 0.45 |
| 09/01/2013 | 10.70 | ± | 0.22 | 17.48 | ± | 0.24 | 12.26 | ± | 0.48 | 14.90 | ± | 0.48 |
| 10/01/2013 | 11.10 | ± | 0.15 | 18.23 | ± | 0.18 | 12.66 | ± | 0.95 | 14.95 | ± | 0.95 |
| 11/01/2013 | 11.67 | ± | 0.26 | 16.83 | ± | 0.32 | 13.26 | ± | 0.80 | 15.48 | ± | 0.80 |
| 12/01/2013 | 11.67 | ± | 0.35 | 17.17 | ± | 0.15 | 13.31 | ± | 0.25 | 14.78 | ± | 0.25 |
| 13/01/2013 | 12.27 | ± | 0.51 | 18.18 | ± | 0.23 | 13.24 | ± | 0.14 | 14.03 | ± | 0.14 |
| 14/01/2013 | 10.43 | ± | 0.37 | 18.40 | ± | 1.11 | 12.84 | ± | 0.80 | 13.43 | ± | 0.80 |
| 15/01/2013 | 10.60 | ± | 0.25 | 14.85 | ± | 0.26 | 12.94 | ± | 0.58 | 12.40 | ± | 0.58 |
| 16/01/2013 | 10.90 | ± | 0.53 | 15.12 | ± | 0.23 | 12.11 | ± | 0.45 | 12.50 | ± | 0.45 |
| 17/01/2013 | 11.83 | ± | 1.33 | 16.14 | ± | 0.23 | 13.11 | ± | 0.38 | 13.15 | ± | 0.38 |
| 18/01/2013 | 10.43 | ± | 0.38 | 17.20 | ± | 0.39 |  | ± |  | 12.90 | ± | 0.07 |
| 19/01/2013 | 10.63 | ± | 0.59 | 16.73 | ± | 0.50 | 12.01 | ± | 0.07 | 12.03 | ± | 0.14 |
| 20/01/2013 | 10.17 | ± | 0.59 | 16.07 | ± | 0.42 | 12.24 | ± | 0.14 | 15.45 | ± | 0.72 |
| 21/01/2013 | 10.73 | ± | 0.28 | 16.28 | ± | 0.30 | 12.01 | ± | 0.72 | 12.95 | ± | 0.25 |
| 22/01/2013 | 10.43 | ± | 0.15 | 15.11 | ± | 0.25 | 12.11 | ± | 0.25 | 13.08 | ± | 0.09 |
| 23/01/2013 | 10.63 | ± | 0.22 | 14.90 | ± | 0.28 | 11.81 | ± | 0.09 | 12.20 | ± | 0.54 |
| 24/01/2013 | 11.00 | ± | 0.41 | 16.47 | ± | 0.58 | 12.77 | ± | 0.54 | 12.93 | ± | 0.18 |
| 25/01/2013 | 11.77 | ± | 0.25 | 16.92 | ± | 0.21 | 13.57 | ± | 0.18 | 14.25 | ± | 0.18 |
| 26/01/2013 | 10.80 | ± | 0.36 | 16.52 | ± | 0.32 | 13.07 | ± | 0.18 | 13.63 | ± | 0.19 |
| 27/01/2013 | 10.33 | ± | 0.26 | 16.31 | ± | 0.21 | 12.77 | ± | 0.19 | 13.53 | ± | 0.47 |
| 28/01/2013 | 10.67 | ± | 0.57 | 16.24 | ± | 0.27 | 12.44 | ± | 0.47 | 13.60 | ± | 0.12 |
| 29/01/2013 | 11.00 | ± | 0.17 | 13.69 | ± | 0.71 | 12.54 | ± | 0.12 | 12.80 | ± | 0.43 |
| 30/01/2013 | 11.27 | ± | 0.49 | 15.22 | ± | 0.10 | 16.01 | ± | 0.43 | 14.83 | ± | 0.22 |
| 31/01/2013 | 10.63 | ± | 0.41 | 15.17 | ± | 0.36 | 14.11 | ± | 0.22 | 15.23 | ± | 0.56 |
| 01/02/2013 | 11.27 | ± | 0.35 | 15.33 | ± | 0.69 | 14.31 | ± | 0.56 | 14.60 | ± | 0.17 |
| 02/02/2013 | 10.77 | ± | 0.55 | 14.87 | ± | 0.24 | 13.04 | ± | 0.17 | 13.65 | ± | 0.58 |
| 03/02/2013 | 10.23 | ± | 0.43 | 14.39 | ± | 0.38 | 12.21 | ± | 0.58 | 14.40 | ± | 0.45 |
| 04/02/2013 | 10.43 | ± | 0.63 | 13.43 | ± | 0.12 | 11.41 | ± | 0.45 | 13.13 | ± | 0.29 |
| 05/02/2013 | 11.10 | ± | 0.45 | 14.72 | ± | 0.37 | 11.21 | ± | 0.29 | 12.88 | ± | 0.45 |
| 06/02/2013 | 10.97 | ± | 0.14 | 15.82 | ± | 0.27 | 11.57 | ± | 0.45 | 13.03 | ± | 0.51 |
| 07/02/2013 | 10.93 | ± | 0.09 | 14.68 | ± | 0.22 | 13.37 | ± | 0.51 | 14.03 | ± | 0.32 |
| 08/02/2013 | 10.70 | ± | 0.38 | 11.46 | ± | 0.34 | 13.97 | ± | 0.32 | 13.68 | ± | 0.28 |
| 09/02/2013 | 11.41 | ± | 0.77 | 13.09 | ± | 0.39 | 12.58 | ± | 0.28 | 13.89 | ± | 0.15 |
| 11/05/2013 | 15.69 | ± | 0.37 | 14.19 | ± | 0.41 | 13.57 | ± | 0.15 | 14.82 | ± | 0.23 |
| 12/05/2013 | 15.16 | ± | 1.22 | 14.96 | ± | 0.25 | 14.39 | ± | 0.07 | 14.76 | ± | 0.18 |
| 13/05/2013 | 14.55 | ± | 0.53 | 15.03 | ± | 0.27 | 14.16 | ± | 0.23 | 1379.35 | ± | 0.16 |
| 14/05/2013 | 14.54 | ± | 0.89 | 14.61 | ± | 0.15 | 13.79 | ± | 0.18 | 14.98 | ± | 0.27 |
| 15/05/2013 | 14.73 | ± | 1.12 | 14.96 | ± | 0.08 | 13.51 | ± | 0.16 | 14.77 | ± | 0.22 |
| 16/05/2013 | 15.63 | ± | 1.00 | 14.78 | ± | 0.16 | 13.99 | ± | 0.27 | 15.70 | ± | 0.28 |
| 17/05/2013 | 14.93 | ± | 0.51 | 13.24 | ± | 0.27 | 13.62 | ± | 0.22 | 15.16 | ± | 0.41 |
| 18/05/2013 | 13.48 | ± | 1.15 | 13.85 | ± | 0.33 | 11.32 | ± | 0.28 | 13.67 | ± | 0.09 |
| 19/05/2013 | 13.81 | ± | 0.81 | 13.71 | ± | 0.14 | 12.71 | ± | 0.41 | 14.42 | ± | 0.24 |
| 20/05/2013 | 13.10 | ± | 0.14 | 14.03 | ± | 0.25 | 12.97 | ± | 0.09 | 14.42 | ± | 0.08 |
| 21/05/2013 | 14.12 | ± | 0.46 | 14.38 | ± | 0.07 | 12.94 | ± | 0.24 | 14.26 | ± | 0.27 |
| 22/05/2013 | 14.22 | ± | 0.54 | 14.50 | ± | 0.06 | 13.68 | ± | 0.08 | 14.46 | ± | 0.15 |
| 23/05/2013 | 14.07 | ± | 0.57 | 14.69 | ± | 0.10 | 14.04 | ± | 0.27 | 15.17 | ± | 0.03 |
| 24/05/2013 | 14.92 | ± | 0.26 | 14.95 | ± | 0.14 | 14.29 | ± | 0.15 | 15.17 | ± | 0.02 |
| 25/05/2013 | 15.06 | ± | 0.46 | 15.05 | ± | 0.07 | 13.77 | ± | 0.03 | 15.18 | ± | 0.04 |
| 26/05/2013 | 15.27 | ± | 0.48 | 14.96 | ± | 0.09 | 13.85 | ± | 0.02 | 14.76 | ± | 0.30 |
| 27/05/2013 | 15.24 | ± | 0.19 | 14.70 | ± | 0.04 | 14.14 | ± | 0.04 | 15.13 | ± | 0.17 |
| 28/05/2013 | 14.85 | ± | 0.33 | 14.77 | ± | 0.12 | 14.42 | ± | 0.30 | 15.81 | ± | 0.24 |
| 29/05/2013 | 14.89 | ± | 0.26 | 14.85 | ± | 0.06 | 14.21 | ± | 0.17 | 15.05 | ± | 0.09 |
| 30/05/2013 | 15.03 | ± | 0.25 | 14.78 | ± | 0.10 | 14.31 | ± | 0.24 | 14.55 | ± | 0.02 |
| 31/05/2013 | 14.52 | ± | 0.20 | 14.32 | ± | 0.04 | 14.36 | ± | 0.09 | 14.40 | ± | 0.03 |
| 01/06/2013 | 14.20 | ± | 0.05 | 14.04 | ± | 0.04 | 14.34 | ± | 0.02 | 14.20 | ± | 0.06 |
| 02/06/2013 | 13.84 | ± | 0.00 | 14.27 | ± | 0.05 | 13.84 | ± | 0.03 | 13.89 | ± | 0.12 |
| 03/06/2013 | 13.88 | ± | 0.24 | 14.50 | ± | 0.07 | 13.79 | ± | 0.06 | 14.20 | ± | 0.10 |
| 04/06/2013 | 13.83 | ± | 0.49 | 14.66 | ± | 0.19 | 14.09 | ± | 0.12 | 14.25 | ± | 0.09 |
| 05/06/2013 | 15.26 | ± | 0.07 | 14.98 | ± | 0.21 | 14.32 | ± | 0.10 | 14.98 | ± | 0.09 |
| 06/06/2013 | 14.30 | ± | 0.64 | 14.85 | ± | 0.07 | 14.37 | ± | 0.09 | 14.70 | ± | 0.03 |
| 07/06/2013 | 14.43 | ± | 0.17 | 14.73 | ± | 0.02 | 14.12 | ± | 0.09 | 14.72 | ± | 0.03 |
| 08/06/2013 | 14.45 | ± | 0.35 | 14.58 | ± | 0.07 | 14.14 | ± | 0.03 | 14.70 | ± | 0.09 |
| 09/06/2013 | 14.38 | ± | 0.32 | 15.02 | ± | 0.29 | 13.89 | ± | 0.03 | 14.93 | ± | 0.02 |
| 10/06/2013 | 14.57 | ± | 0.19 | 14.86 | ± | 0.04 | 14.05 | ± | 0.09 | 14.83 | ± | 0.08 |
| 11/06/2013 | 14.90 | ± | 0.08 | 14.22 | ± | 0.15 | 13.98 | ± | 0.02 | 14.11 | ± | 0.01 |
| 12/06/2013 | 15.07 | ± | 0.01 | 15.00 | ± | 0.12 | 14.20 | ± | 0.08 | 14.80 | ± | 0.03 |
| 13/06/2013 | 14.77 | ± | 0.09 | 14.96 | ± | 0.16 | 14.36 | ± | 0.01 | 14.40 | ± | 0.17 |
| 14/06/2013 | 14.71 | ± | 0.10 | 14.78 | ± | 0.19 | 14.38 | ± | 0.03 | 14.21 | ± | 0.17 |
| 15/06/2013 | 14.68 | ± | 0.27 | 14.54 | ± | 0.28 | 14.22 | ± | 0.17 | 14.49 | ± | 0.29 |
| 16/06/2013 | 14.54 | ± | 0.13 | 14.17 | ± | 0.27 | 14.07 | ± | 0.17 | 14.38 | ± | 0.10 |
| 17/06/2013 | 14.58 | ± | 0.05 | 14.96 | ± | 0.14 | 13.80 | ± | 0.29 | 13.59 | ± | 0.11 |
| 18/06/2013 | 14.59 | ± | 0.01 | 14.71 | ± | 0.13 | 13.98 | ± | 0.10 | 13.90 | ± | 0.15 |
| 19/06/2013 | 14.59 | ± | 0.01 | 15.03 | ± | 0.12 | 14.20 | ± | 0.11 | 14.15 | ± | 0.18 |
| 20/06/2013 | 14.48 | ± | 0.09 | 14.68 | ± | 0.09 | 14.27 | ± | 0.15 | 13.75 | ± | 0.48 |
| 21/06/2013 | 14.55 | ± | 0.27 | 14.56 | ± | 0.28 | 14.17 | ± | 0.18 | 13.39 | ± | 0.17 |
| 22/06/2013 | 14.57 | ± | 0.17 | 14.96 | ± | 0.25 | 14.10 | ± | 0.48 | 13.78 | ± | 0.02 |
| 23/06/2013 | 14.22 | ± | 0.08 | 14.45 | ± | 0.16 | 14.36 | ± | 0.17 | 14.23 | ± | 0.08 |
| 24/06/2013 | 14.47 | ± | 0.08 | 14.67 | ± | 0.13 | 14.12 | ± | 0.02 | 13.65 | ± | 0.08 |
| 25/06/2013 | 14.43 | ± | 0.31 | 15.31 | ± | 0.51 | 14.18 | ± | 0.08 | 13.63 | ± | 0.13 |
| 26/06/2013 | 14.63 | ± | 0.28 | 14.78 | ± | 0.22 | 14.30 | ± | 0.08 | 14.15 | ± | 0.01 |
| 27/06/2013 | 14.42 | ± | 0.21 | 14.39 | ± | 0.46 | 14.22 | ± | 0.13 | 14.68 | ± | 0.07 |
| 28/06/2013 | 14.31 | ± | 0.08 | 14.39 | ± | 0.15 | 14.03 | ± | 0.01 | 14.90 | ± | 0.03 |
| 29/06/2013 | 14.37 | ± | 0.21 | 14.30 | ± | 0.16 | 14.07 | ± | 0.07 | 13.80 | ± | 0.13 |
| 30/06/2013 | 13.99 | ± | 0.13 | 14.43 | ± | 0.24 | 13.94 | ± | 0.03 | 13.61 | ± | 0.07 |
| 01/07/2013 | 13.75 | ± | 0.08 | 14.57 | ± | 0.24 | 13.80 | ± | 0.13 | 13.60 | ± | 0.17 |
| 02/07/2013 | 13.08 | ± | 0.14 | 15.00 | ± | 0.18 | 13.61 | ± | 0.07 | 13.17 | ± | 0.31 |
| 03/07/2013 | 12.60 | ± | 0.16 | 15.11 | ± | 0.35 | 13.41 | ± | 0.17 | 13.54 | ± | 0.06 |
| 04/07/2013 | 12.89 | ± | 0.24 | 14.28 | ± | 0.15 | 13.05 | ± | 0.31 | 14.48 | ± | 0.10 |
| 05/07/2013 | 13.92 | ± | 0.12 | 14.21 | ± | 0.10 | 13.18 | ± | 0.06 | 13.66 | ± | 0.03 |
| 06/07/2013 | 14.35 | ± | 0.01 | 14.25 | ± | 0.08 | 13.49 | ± | 0.10 | 13.98 | ± | 0.06 |
| 07/07/2013 | 14.46 | ± | 0.16 | 15.11 | ± | 0.23 | 13.58 | ± | 0.03 | 13.96 | ± | 0.12 |
| 08/07/2013 | 14.66 | ± | 0.19 | 15.08 | ± | 0.19 | 13.66 | ± | 0.06 | 13.94 | ± | 124.15 |
